# Supplementary material for: Prioritizing non-coding regions based on human genomic constraint and sequence context with deep learning
Source: Nat Commun. 2021 Mar 8;12:1504. doi: 10.1038/s41467-021-21790-4 (PMC7940646; doi:10.1038/s41467-021-21790-4)
Supplement: Supplementary file 1 — Supplementary Information [file 41467_2021_21790_MOESM1_ESM.pdf]

Supplementary Information

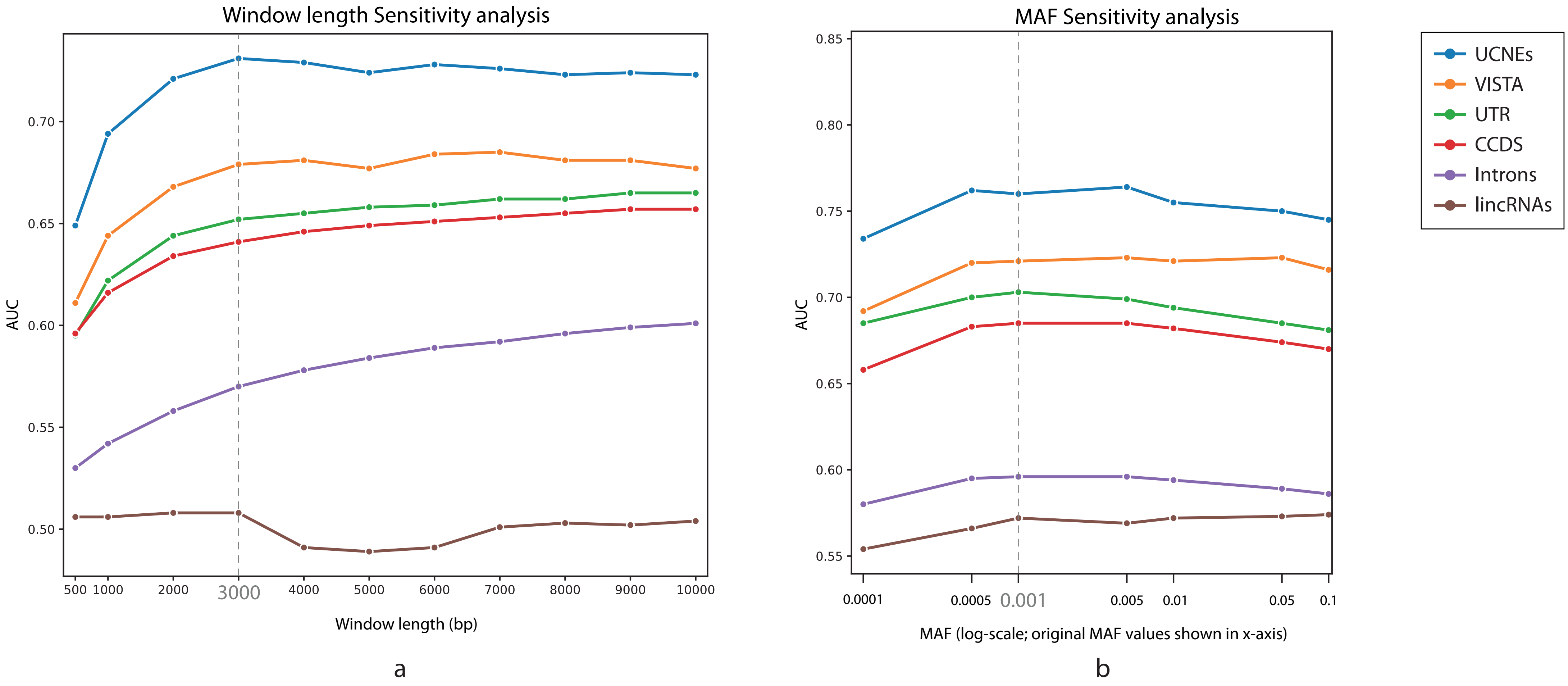

Supplementary Figure 1. Sensitivity analysis for optimal selection of a) window length and b) Minor Allele Frequency (MAF) threshold for gwRVIS calculation. The output examined in this sensitivity analysis is the segregation of non-intergenic genomic classes compared to the intergenic class. This is quantified by the AUC score of a Logistic Regression classifier, trying to distinguish sets of windows from two different genomic classes using 10-fold Cross-Validation. The aim of the analysis is to achieve the best possible segreggation with the smallest possible window length. A 3kb window length has been selected in this case and then a further MAF sensitivity analysis has been performed for 8 different values: 0.0001, 0.0005, 0.001, 0.005, 0.01, 0.05, 0.1, 0.5. We have selected MAF=0.001 as our default value, as it achieves the peak of AUC performance in the majority of genomic classes (along with 0.005 and 0.01).

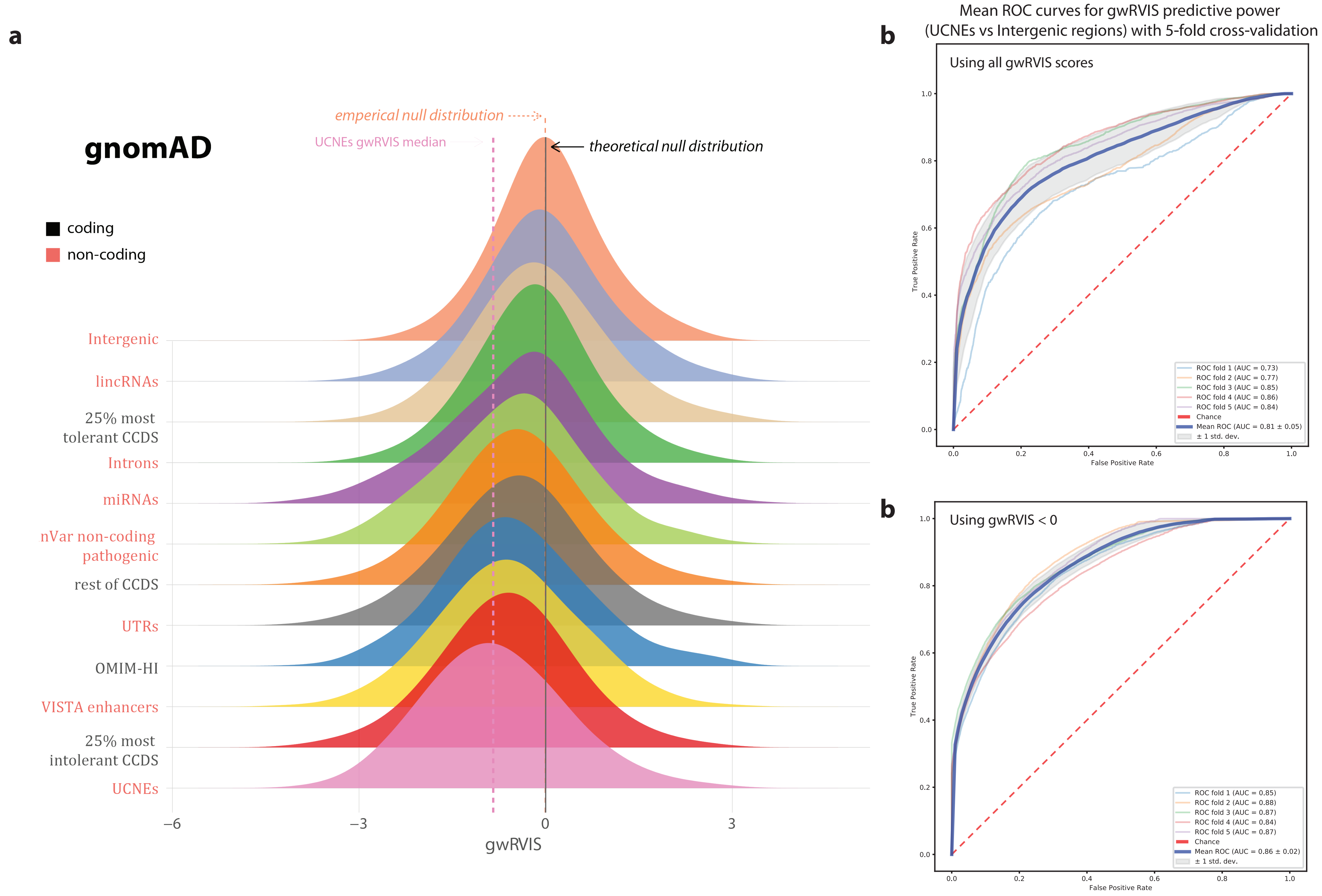

Supplementary Figure 2. Fine-grain genome-wide performance of gwRVIS. **a.** Distribution of gwRVIS scores across 12 genomic classes, in descending order of intolerance to variation: intergenic regions, lincRNAs, 25% most tolerant CCDS (based on RVIS), introns, miRNAs, ClinVar non-coding pathogenic, rest of CCDS (25-75 percentile based on RVIS), UTRs, OMIM Haploinsufficient, VISTA enhancers, 25% most intolerant CCDS (based on RVIS) and UCNEs (Ultra Conserved Non-Coding Elements).
b. 5-fold cross-validation for classification of UCNE-vs-Intergenic positions with gwRVIS, when including all gwRVIS scores. **c.** 5-fold cross-validation for classification of UCNE-vs-Intergenic positions with gwRVIS, when including only genomic regions with negative selection (gwRVIS < 0). A logistic regression model has been employed in both **(b)** and **(c)**.

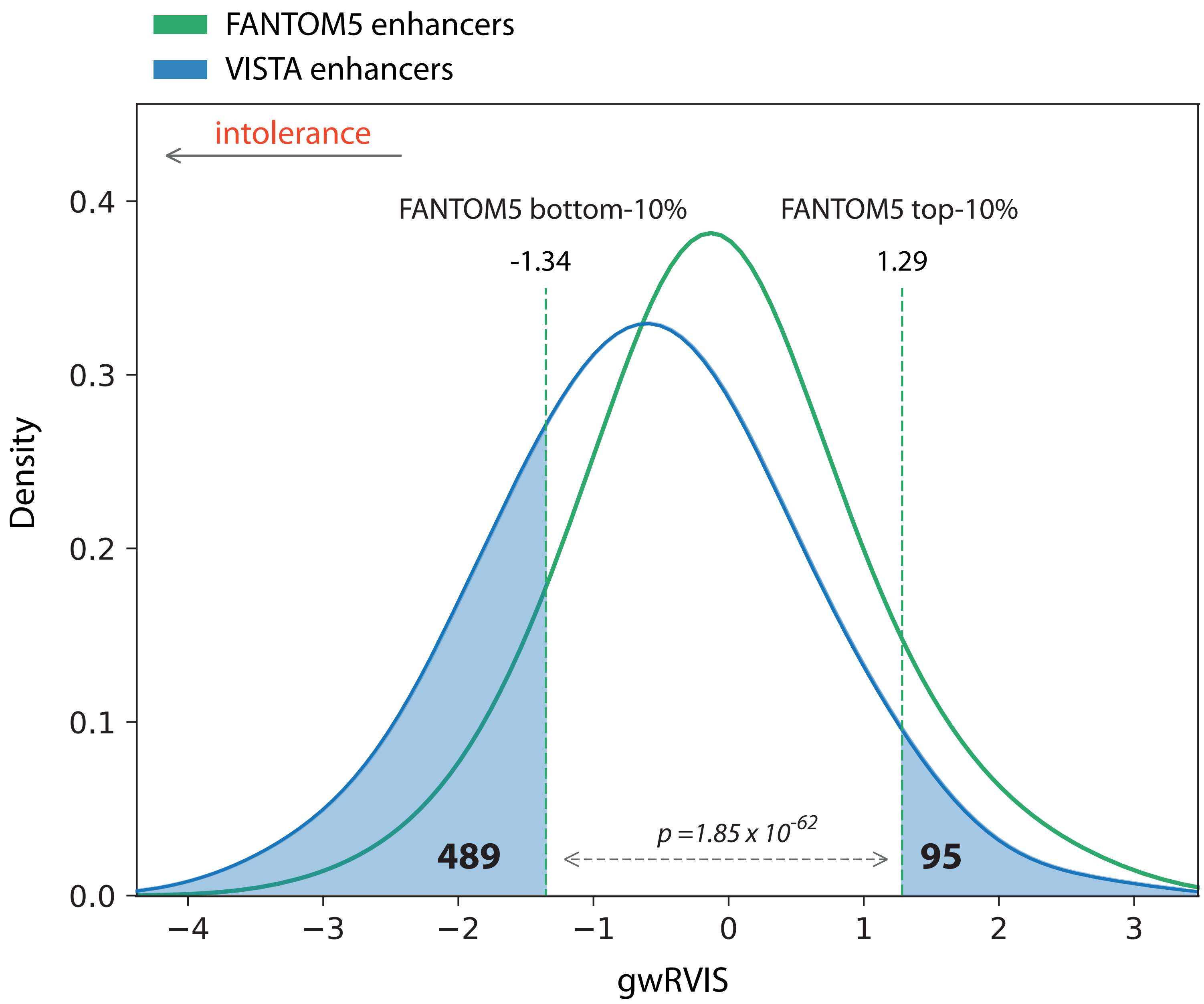

Supplementary Figure 3. Distribution of VISTA enhancers across all FANTOM5 enhancers: the 10% most intolerant FANTOM5 enhancers were significantly enriched for VISTA enhancers compared to the 10% most tolerant ones (489 vs 95 windows; 5.15-fold increase, two-sided Fisher's exact test $p = 1.85\times10^{-62}$).

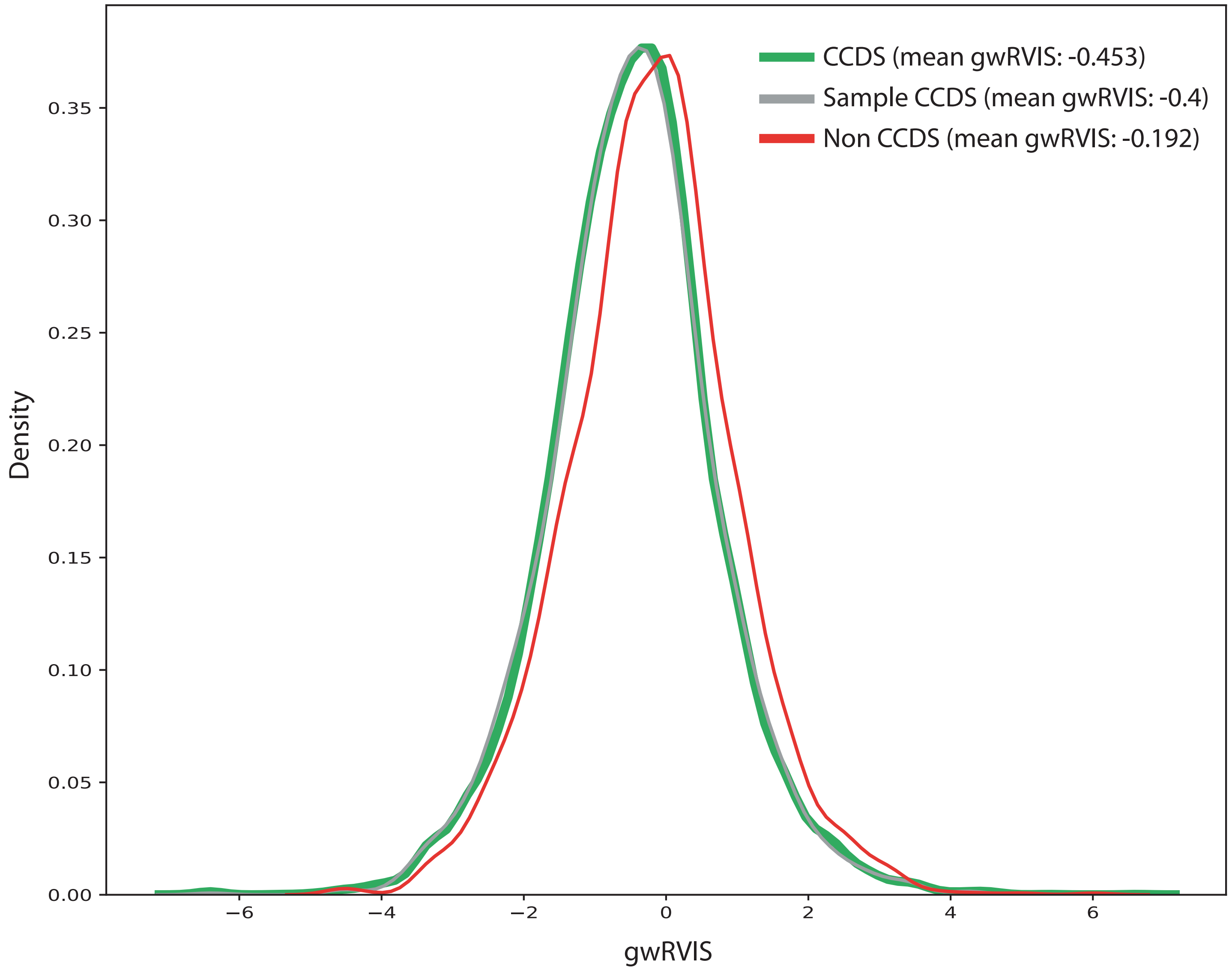

Supplementary Figure 4. gwRVIS benchmarking using a set of 996 CCDS and 989 non-CCDS regions from the Orion paper (Gussow et al., 2017). The segregation between CCDS and non-CCDS regions achieved by gwRVIS is significantly greater than the one achieved by Orion: $p= 8.1x\ 10^{-23}$ for gwRVIS while p=0.001 for Orion (two-sided Mann-Whitney U test). A similar significant segregation is also achieved when comparing all non-CCDS regions against a matched sample of CCDS regions of equal size: p-value = 8.44×10^{-15} (two-sided Mann-Whitney U test).

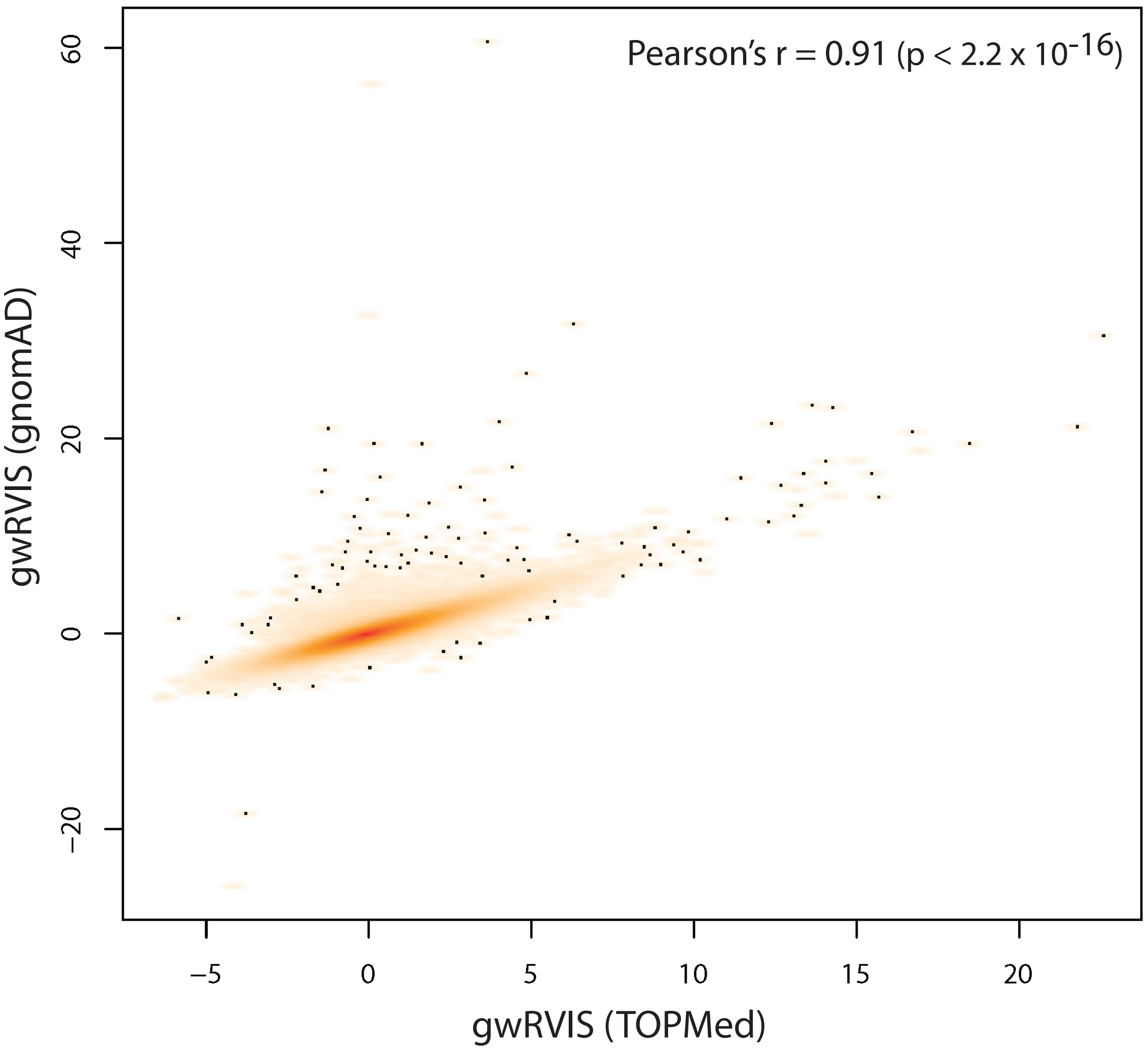

Supplementary Figure 5. gwRVIS consistency on two different WGS datasets: Correlation plot between TOPMed- and gnomAD-derived gwRVIS values; Pearson's correlation coefficient: r = 0.91, p < 2.2 x 10-16.

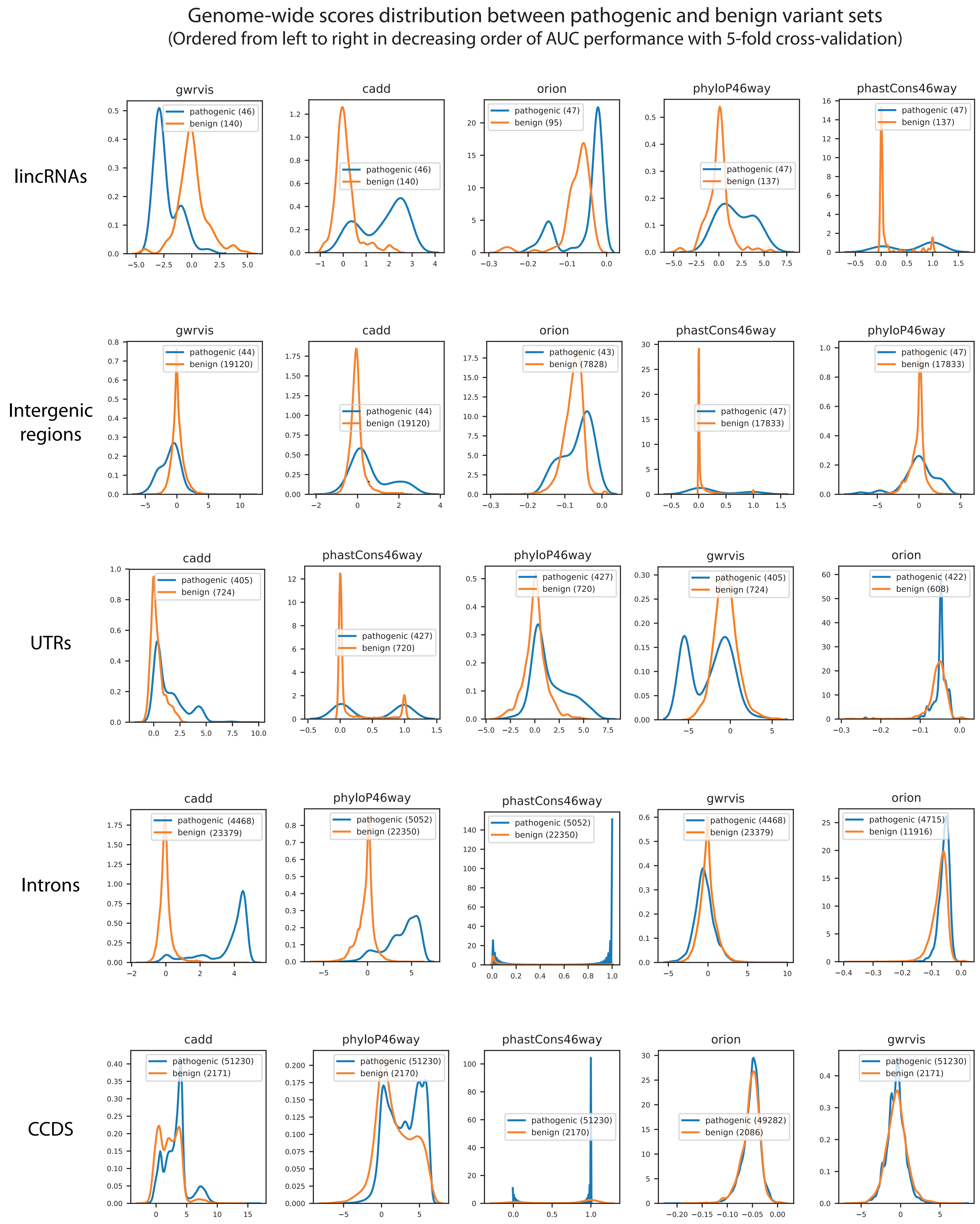

Supplementary Figure 6. Distribution of genome-wide scores between pathogenic and benign sets of variant loci across five different genomic classes: lincRNAs, intergenic, UTRs, Introns and CCDS. Pathogenic variants are derived from ClinVar while benign variants have been extracted from denovo-db, as those annotated with a "control" primary phenotype. For each genomic class, scores are sorted from left to right in decreasing order of AUC performance achieved by a 5-fold Logistic Regression classifier. For this classification task, class imbalance has been resolved by sampling a random sample from the benign set mathcing the respective pathogenic set in size.

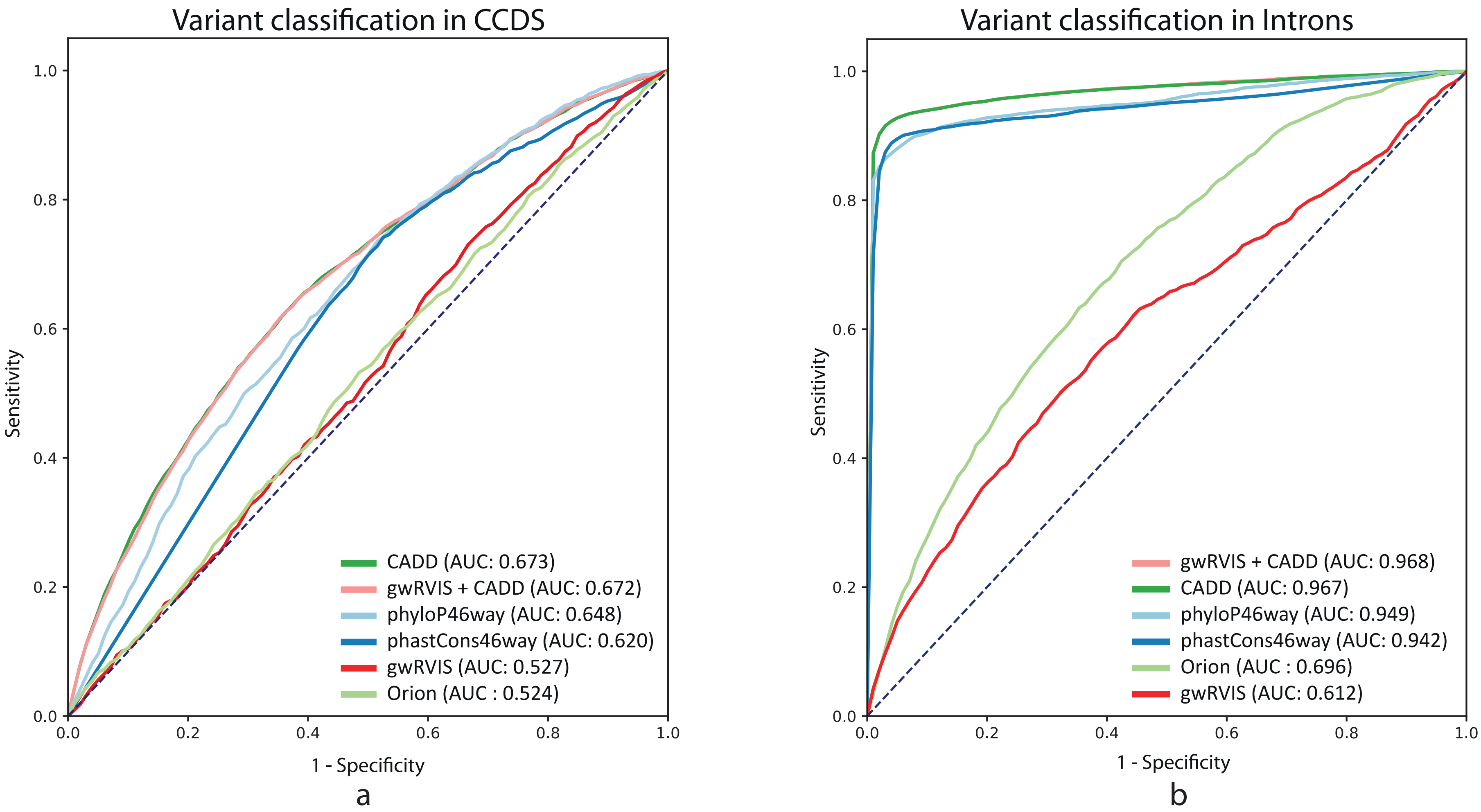

Supplementary Figure 7. Benchmarking of genome-wide scores for pathogenic-vs-benign variant classification. Mean ROC curves from 5-fold Cross-Validation prediction with Logistic Regression are shown across two genomic classes: a) CCDS and b) Introns.

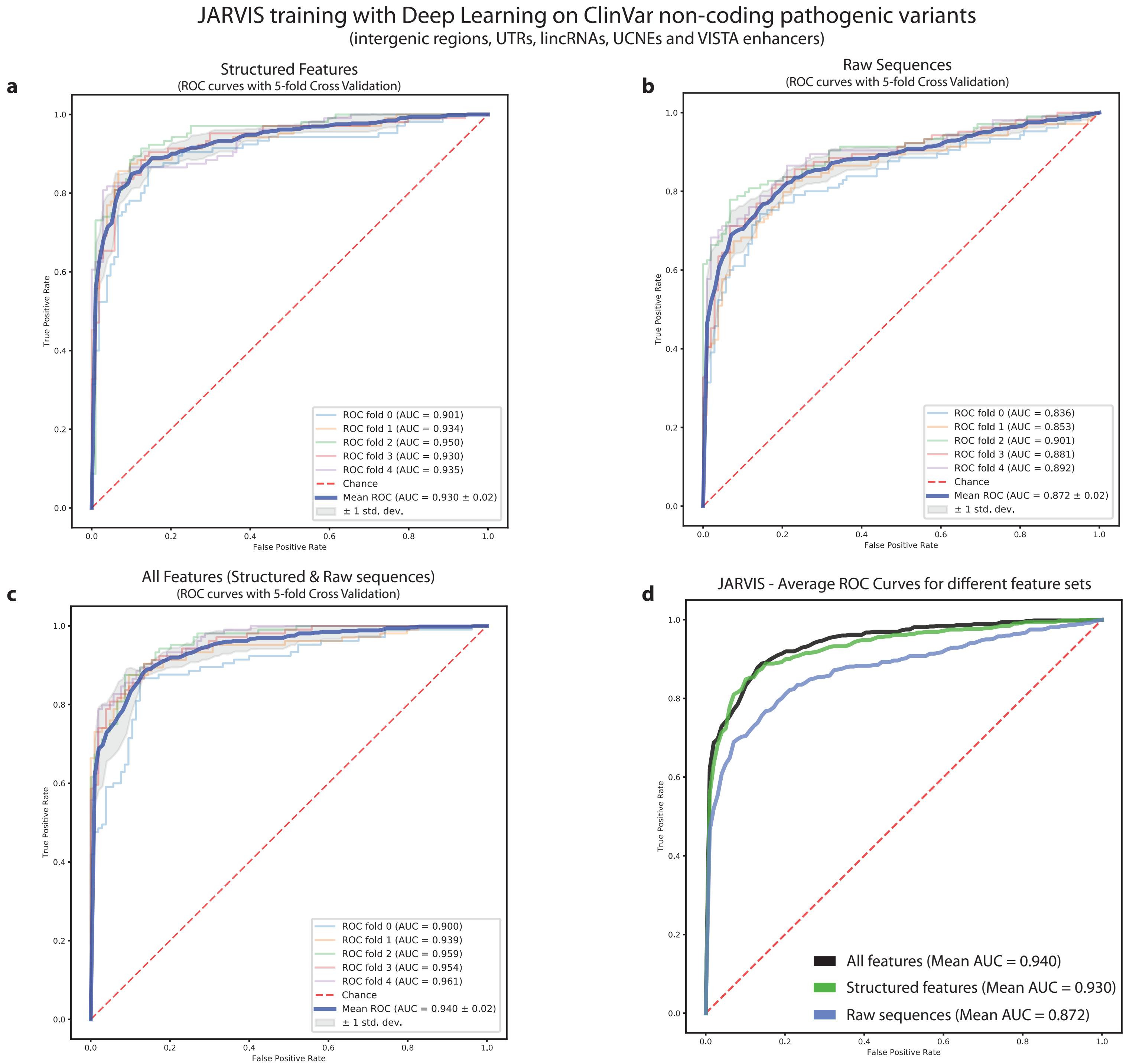

Supplementary Figure 8. JARVIS training with Deep Learning using different feature sets. ROC Curves with 5-fold Cross-Validation using: **a)** Structured features, **b)** Raw Sequences, and **c)** All features (Structured and Raw sequences). **d)** Mean ROC curves from JARVIS training with deep neural networks using the three different feature set combinations. Training has been performed using all 521 pathogenic variants annotated in ClinVar for the full non-coding model (i.e. encompassing intergenic regions, UTRs, lincRNAs, UCNEs, or Vista enhancers) and a negative set of benign variants of equal size extracted randomly from a set of "control" variants derived from denovo-db. Mean ROC in all plots refers to the ROC of the mean of model predictions from each cross-validation split.

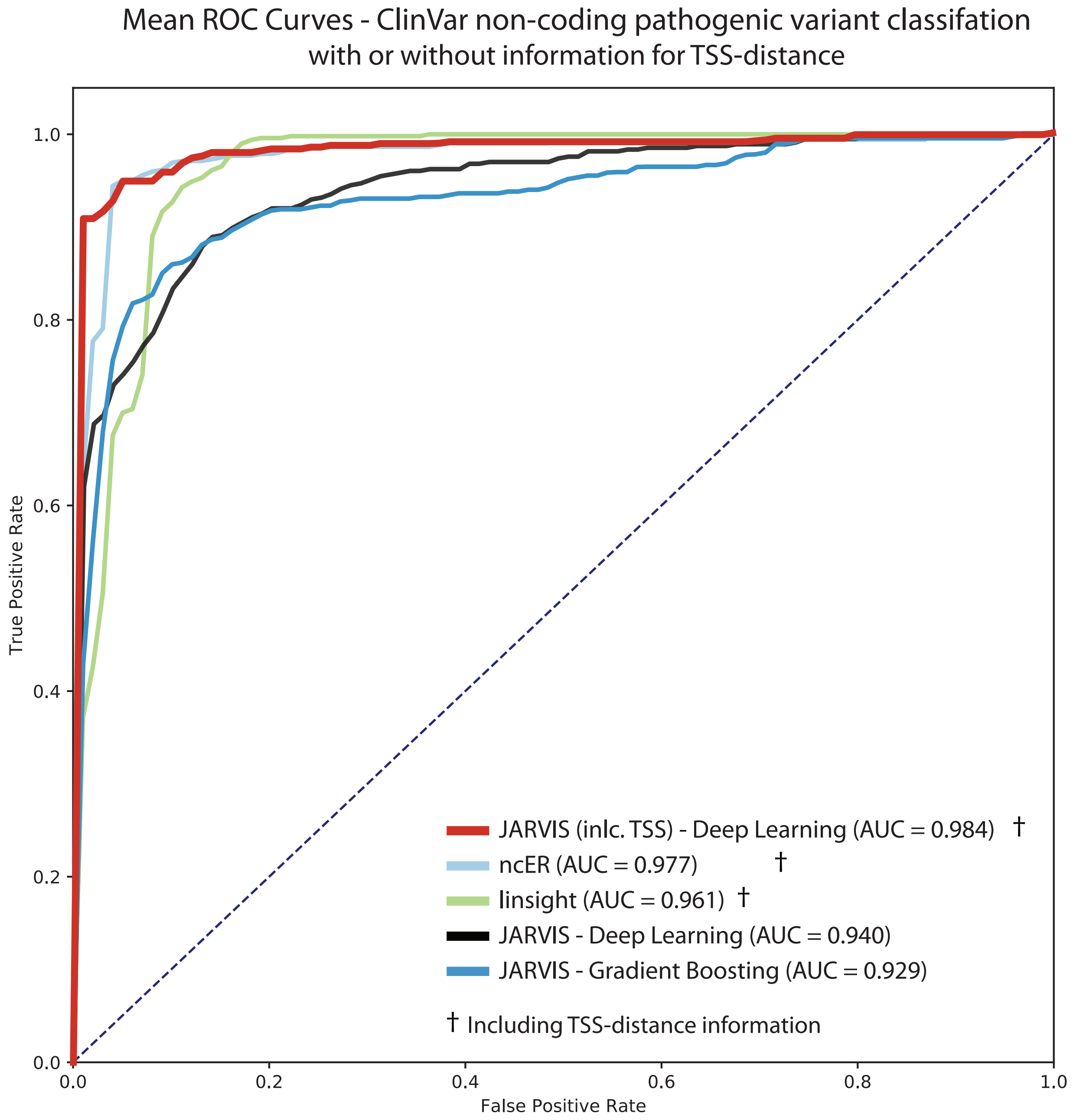

Supplementary Figure 9. Mean ROC curves from ClinVar non-coding pathogenic variants classification (intergenic regions, UTRs, lincRNAs, UCNEs and VISTA enhancers) with 5-fold Cross Validation. ncER and LINSIGHT integrate by default the TSS-distance information in their construction. Three models have been trained for JARVIS: 1) the default one employing the structured features and raw sequences (black color), 2) the model trained by Gradient Boosting using only the structured features (dark blue color) and a JARVIS version based on structured featrues that also includes the TSS-distance information (red color), outperforming all other scores in the ClinVar training set.

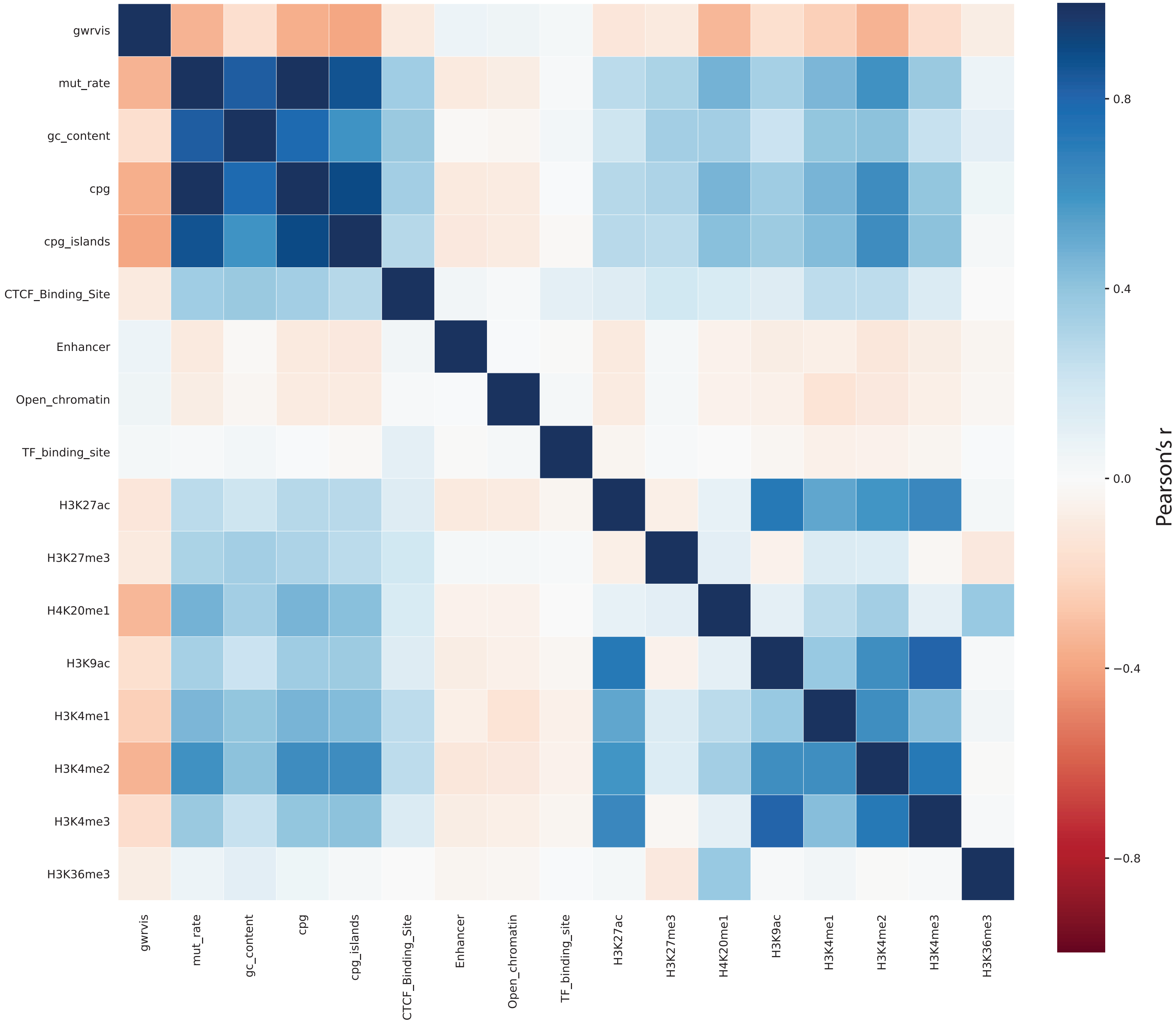

Supplementary Figure 10. Pearson's correlations between all pairs of structured features integrated within the JARVIS model.

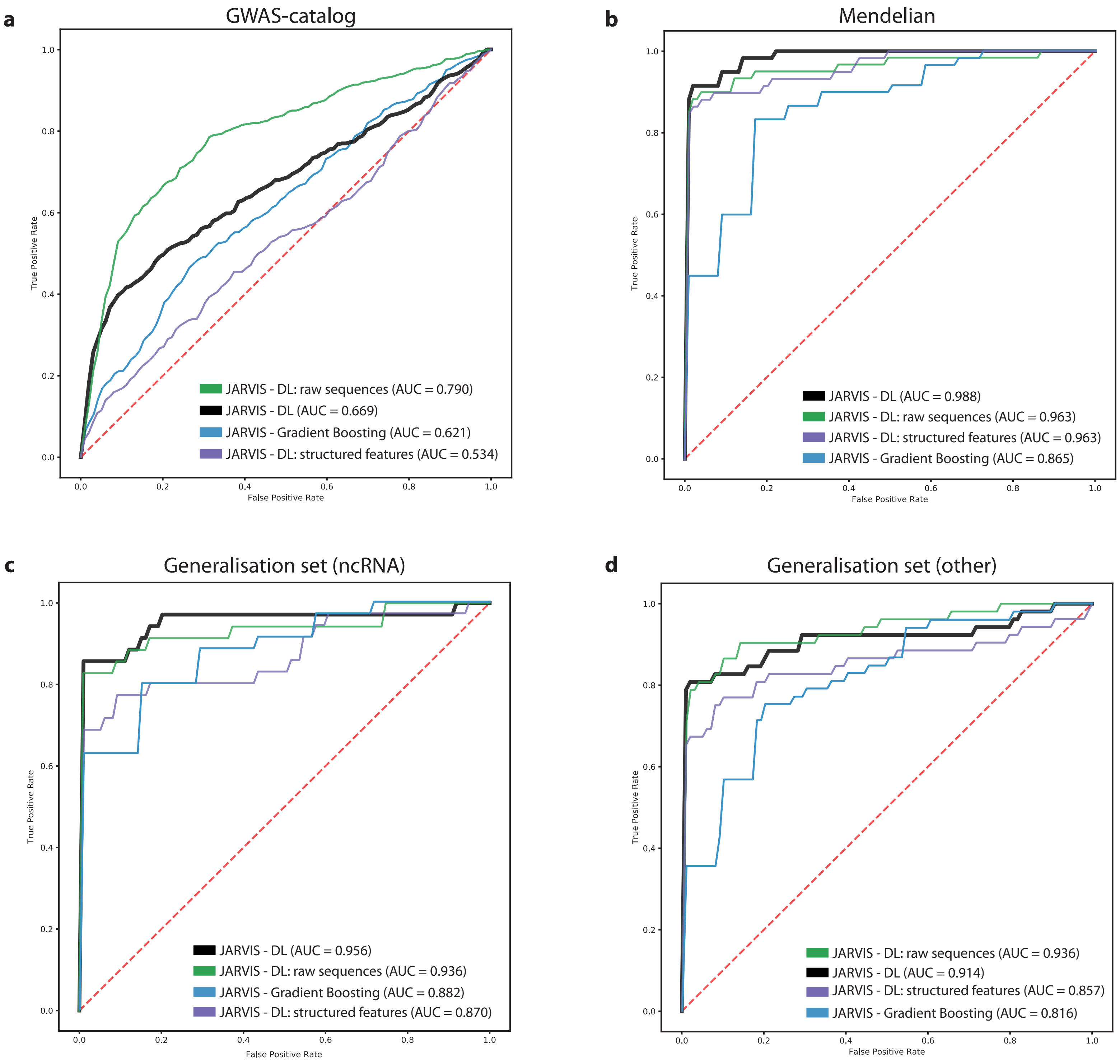

Supplementary Figure 11. JARVIS performance on four independent test sets (a-d) based on different training models.

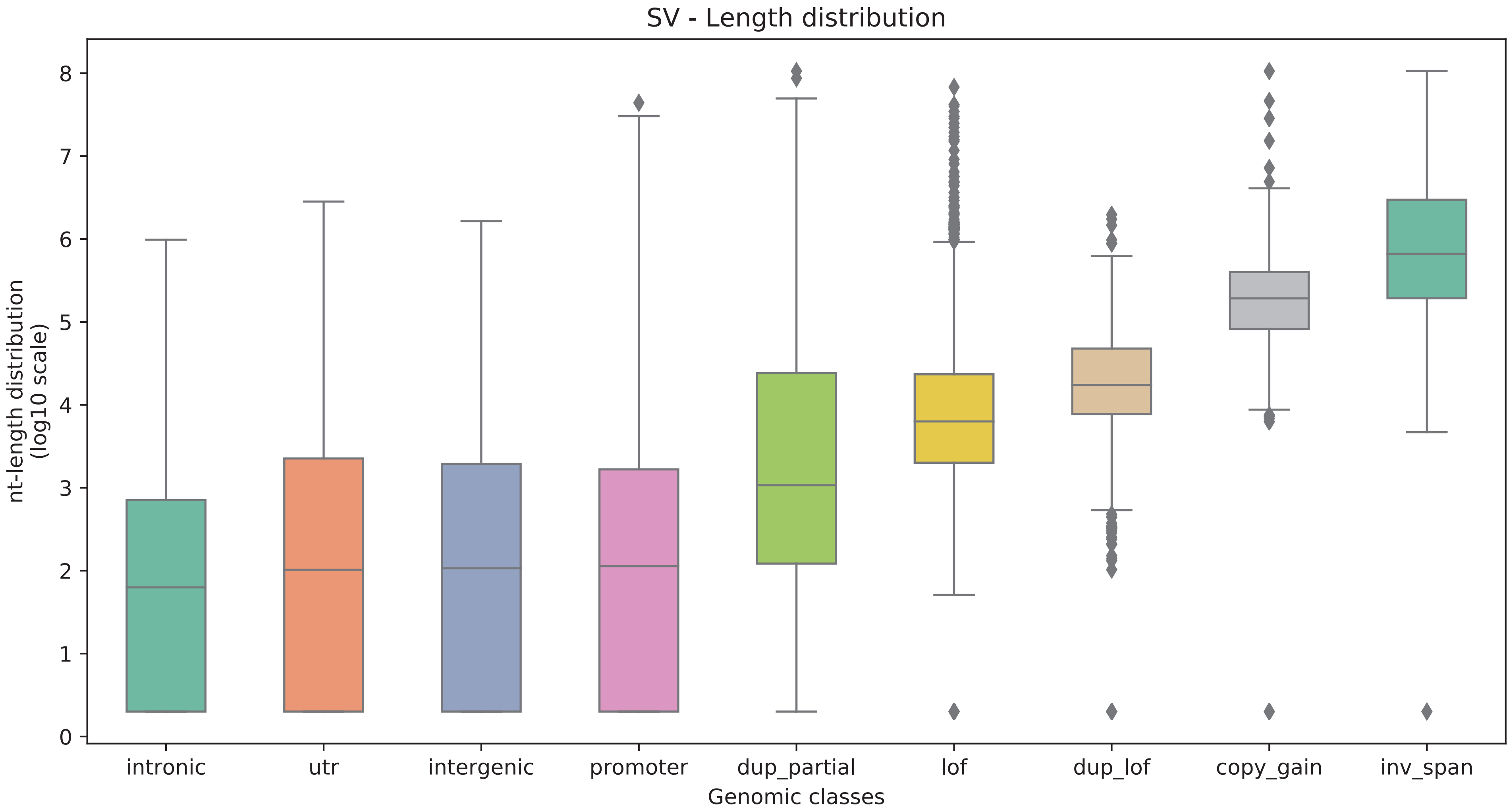

Supplementary Figure 12. Length distribution (log scale) across different types of structural variants from gnomAD. For each boxplot, its central line represents the median, the bounds represent the 25th and 75th percentile, and the whiskers extend up to 1.5 the interquartile range from the respective bounds.

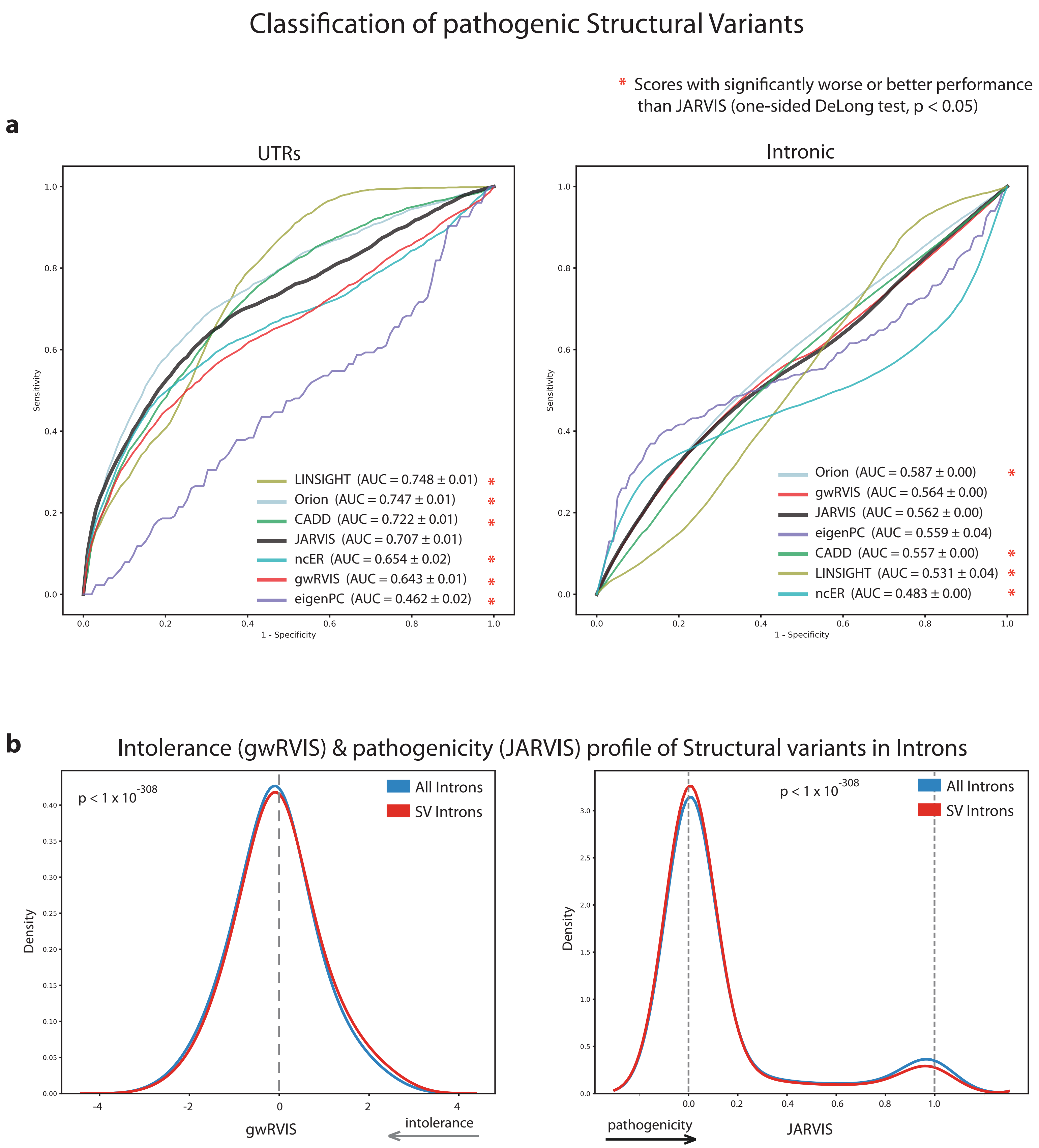

Supplementary Figure 13. JARVIS and gwRVIS performance on structural variants. **a)** ROC curves from classification of benign structural variants (intergenic) against different sets of putative pathogenic ones from gnomAD, annotated as UTRs or Intronic. A 10-fold cross-validation approach with a logistic regression model has been used across five scores: JARVIS, gwRVIS, LINSIGHT, ncER and Orion. **b)** gwRVIS and JARVIS distributions across the entire intronic genomic class and a set of called structural variants with intronic annotation in gnomAD. Intolerance increases towards lower gwRVIS values while pathogenicity likelihood increases with greater JARVIS values.

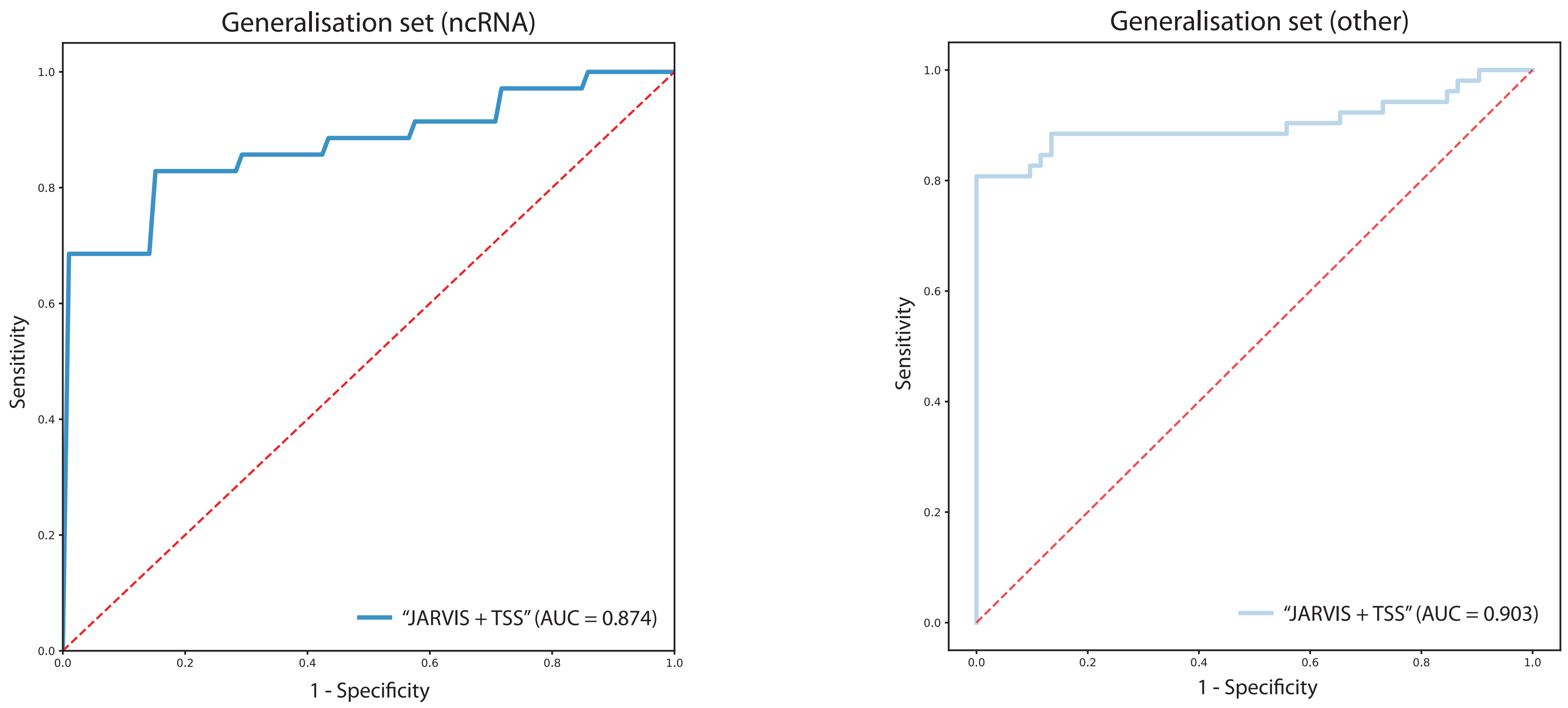

Supplementary Figure 14. JARVIS performance on two test sets after having been trained with the TSS distance as an additional feature in the default multi-module deep learning network.

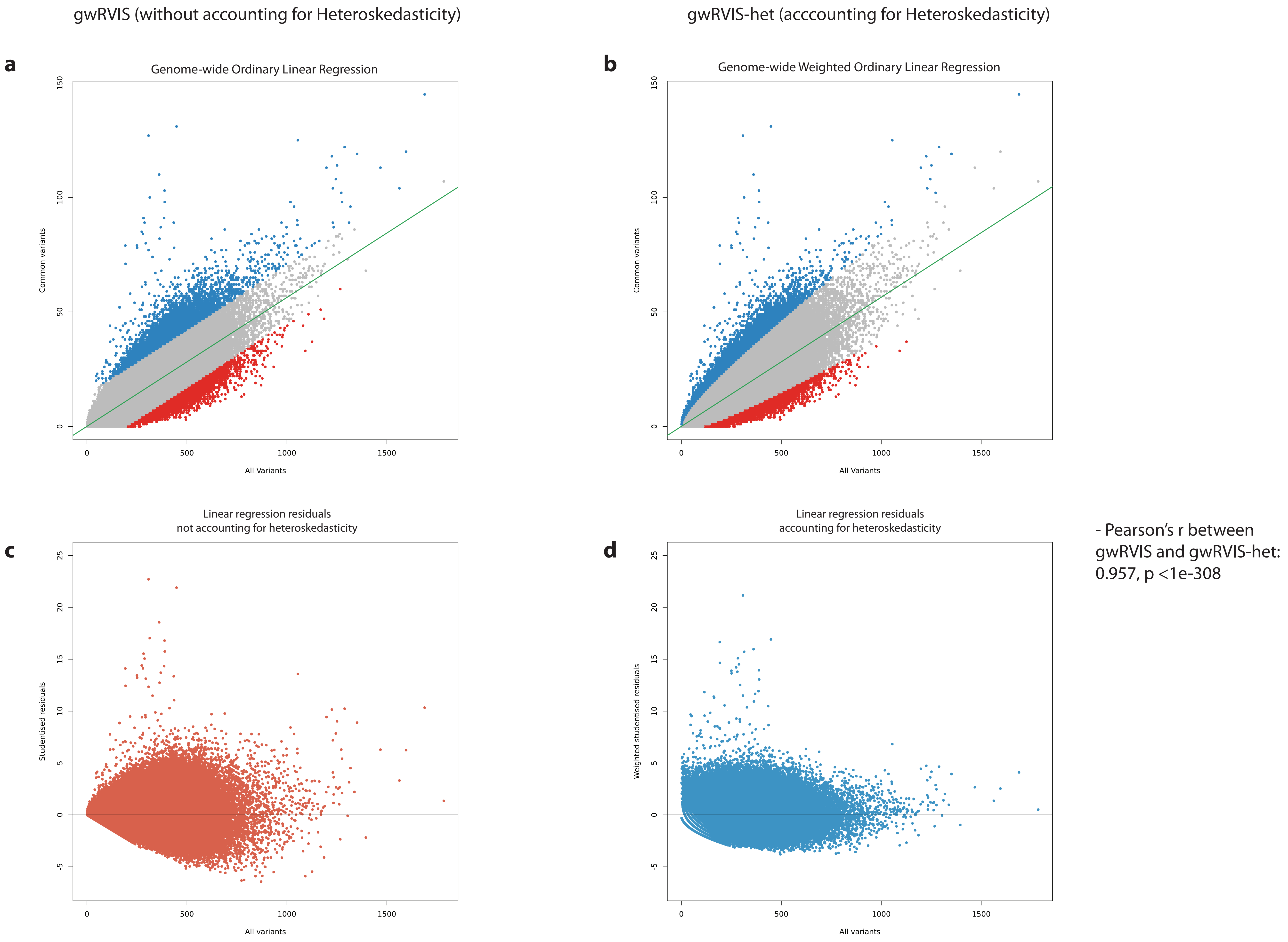

Supplementary Figure 15. gwRVIS calculation with or without accounting for heteroskedasticity. **a)** An ordinary linear regression model is fit between "all" and "common" (MAF > 0.1%) variants across all windows. **b)** A weighted ordinary linear regression model is fit, accounting for the heteroskedasticity observed in the windows with lower values of "all variants". gwRVIS is shown in **c)** and **d)** for the two models respectively. Pearson's correlation between the two versions of gwRVIS calculations is 0.957 (p <1e-308; two-sided t-test).

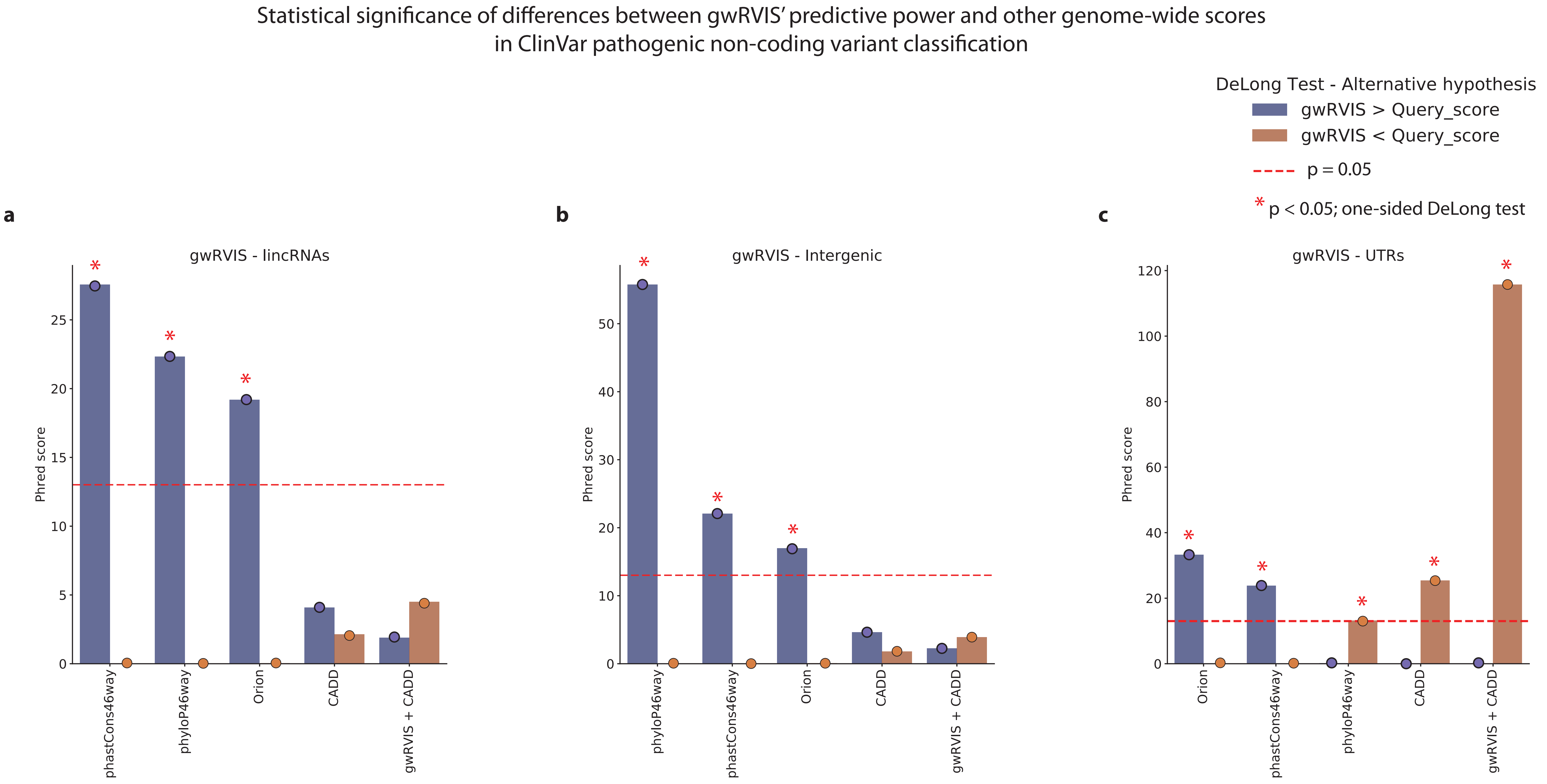

Supplementary Figure 16. Statistical significance of the differences in gwRVIS predictive power against four other genome-wide scores and a combined model integrating CADD & gwRVIS, using one-sided DeLong's tests for both alternative hypotheses (i.e. "less" or "greater"). The predictive power of each score is assessed via the average AUC score attained with 5-fold cross-validation during ClinVar-pathogenic vs denovodb-benign variants classification. Results are shown separately for the classification task across three non-coding genomic classes: **a)** lincRNAs, **b)** Intergenic regions and **c)** UTRs, and are visualised as Phred scores. The red dashed horizontal line marks the p = 0.05 significance threshold [y=Phred(0.05)]. Barplots in light puhrple colour that exceed the significance threshold of Phred(0.05) support that gwRVIS significantly outperforms the respective score (gwRVIS > Query_score). On the other hand, barplots in orange colour that exceed the significance threshold of Phred(0.05) support that gwRVIS significantly *underperforms* the respective score (gwRVIS < Query_score). Raw p-values from each DeLong test are available at Supplementary Tables 1-3.

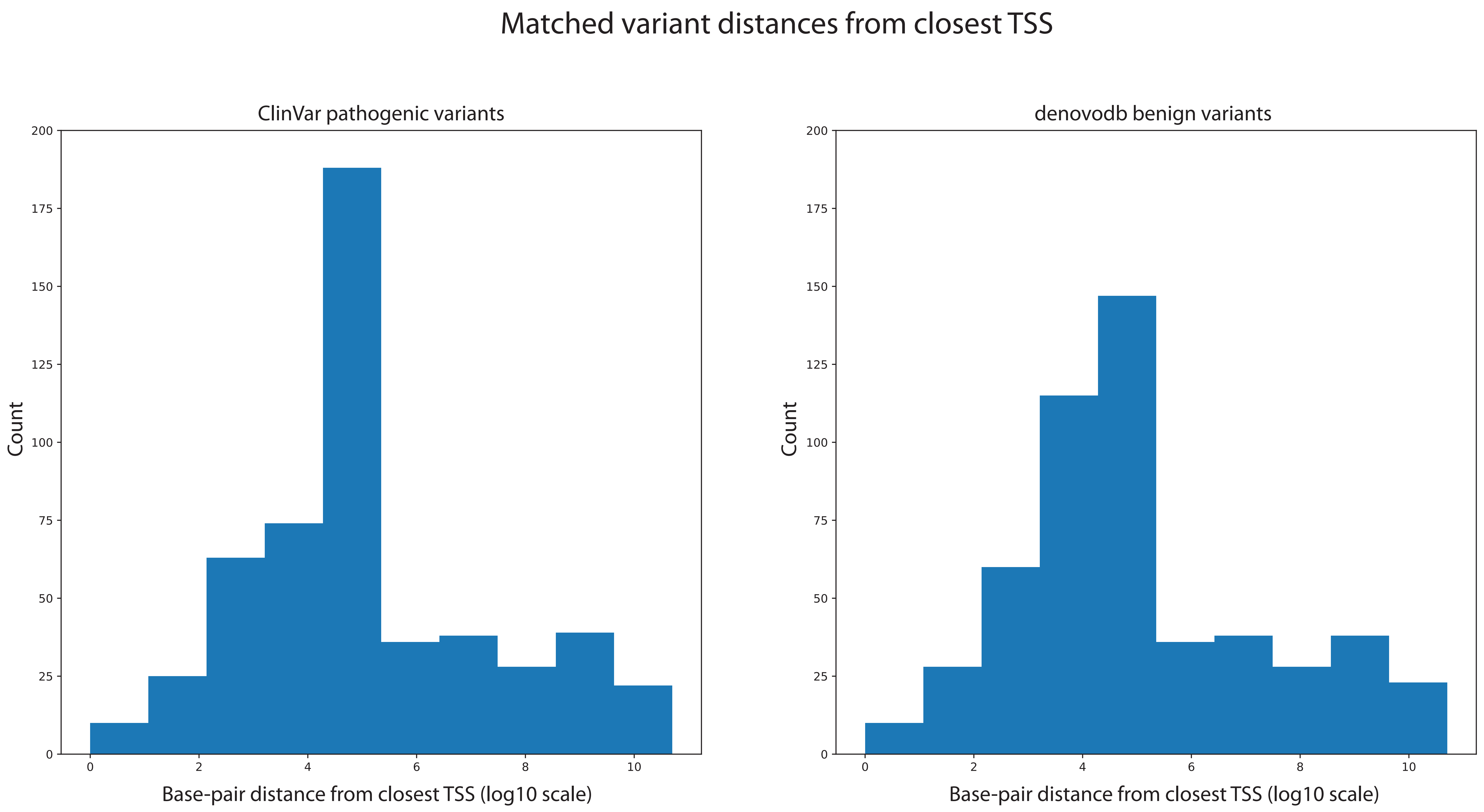

Supplementary Figure 17. Distribution of matched variant distances from closest TSS. **a)** ClinVar pathogenic variants **b)** denovo-db benign variants.

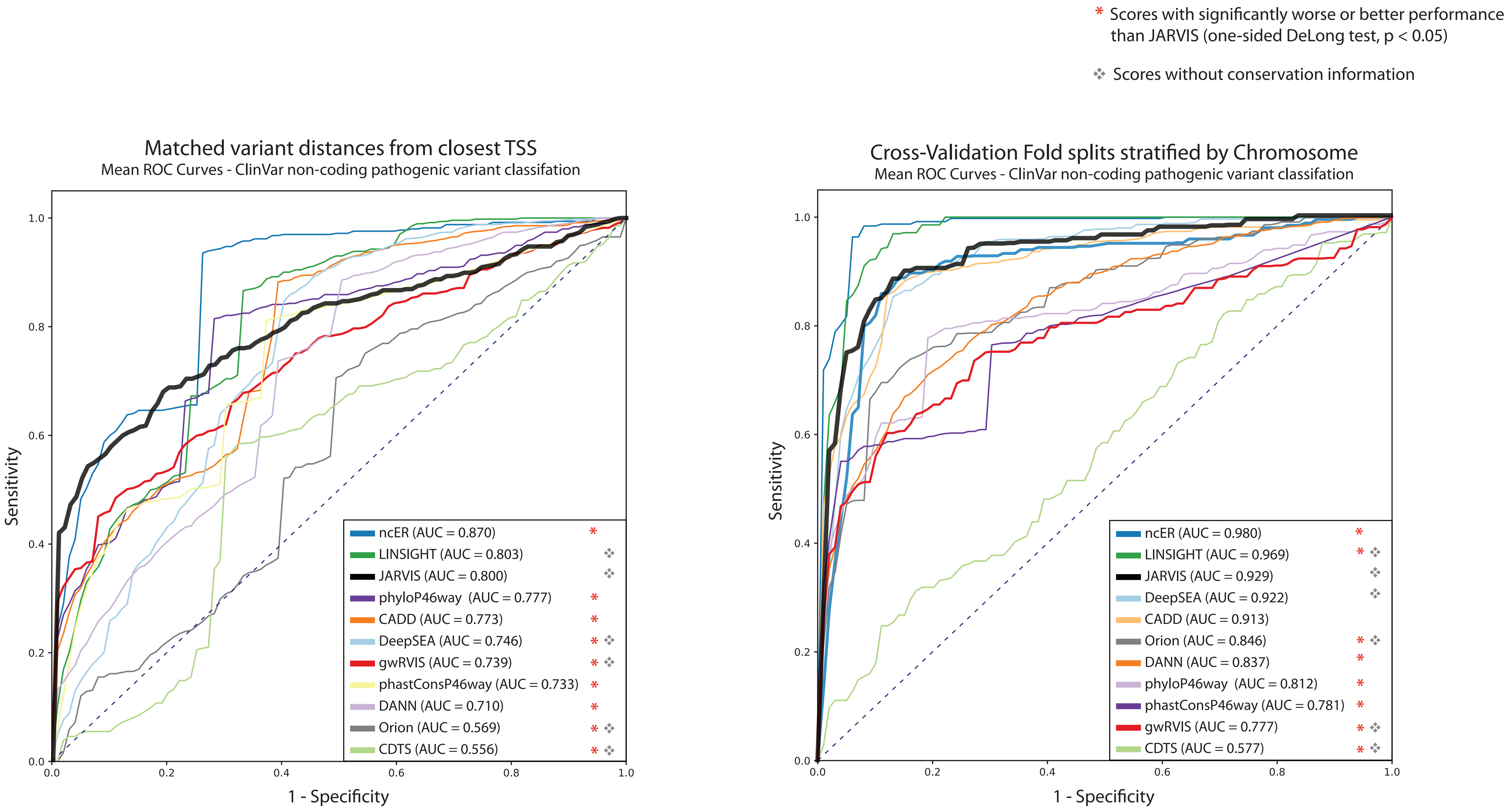

Supplementary Figure 18. JARVIS 5-fold cross-validation training performance using two alternative strategies: **a)** training set of ClinVar pathogenic and denovodb benign variants matched in terms of their distances to closest TSS and **b)** cross-validation splits stratified by chromosome, to ensure that, at each cross-validation step, variants from the same chromosomes are not part of the training and test set at the same time. A set of 10 other genome-wide scores have been tested with 5-fold cross-validation on the same datasets (eigenPC did not have sufficient number of data points using these strategies and thus is omitted from these comparisons).

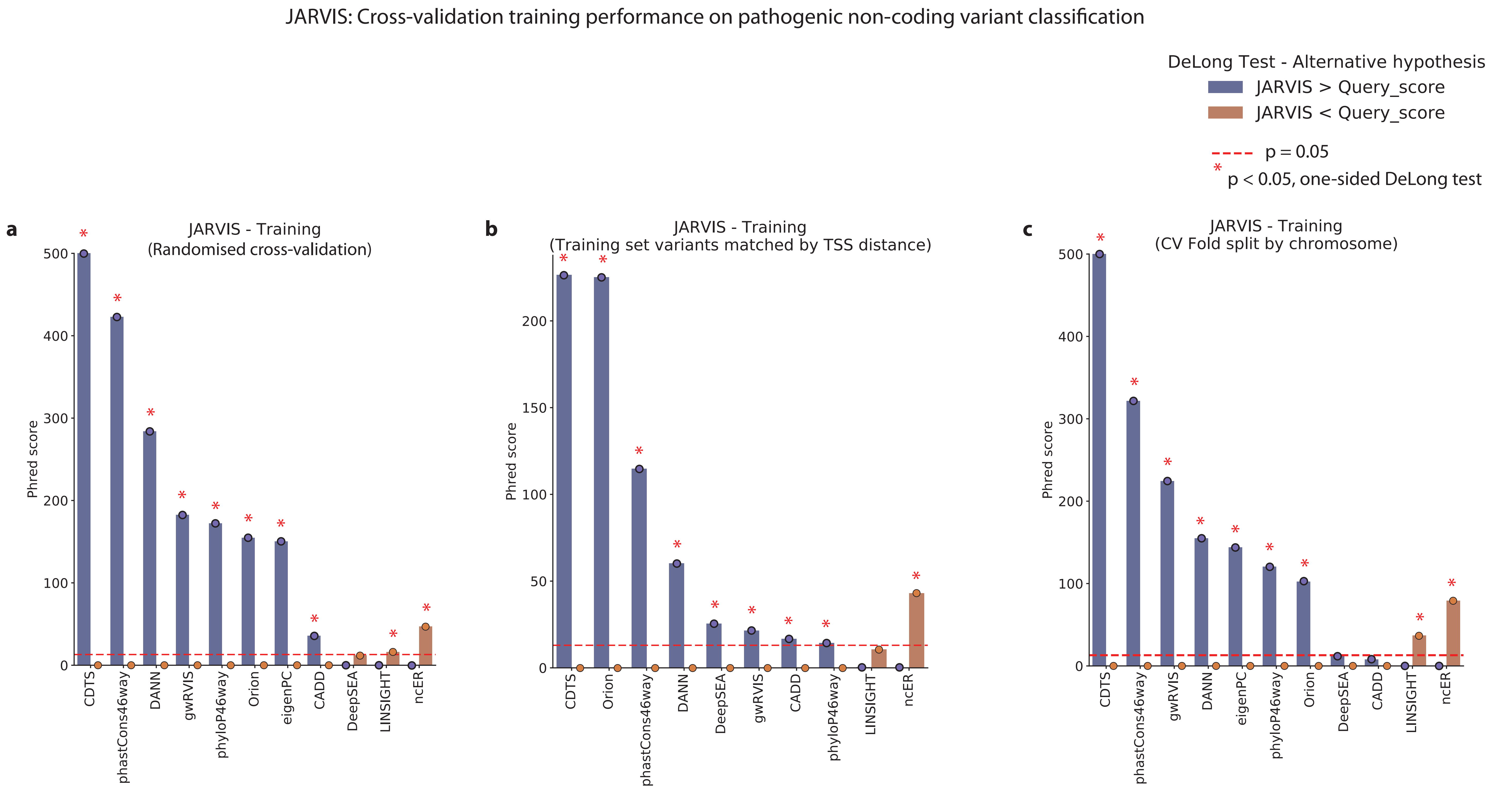

Supplementary Figure 19. Statistical significance of the differences in JARVIS predictive power against 11 other genome-wide scores using one-sided DeLong's tests for both alternative hypotheses (i.e. "less" or "greater"). The predictive power of each score is assessed via the average AUC score attained with 5-fold cross-validation during ClinVar-pathogenic vs denovodb-benign non-coding variant classification. DeLong's test results are visualised as Phred scores (scores > 500 are capped at this value for visualisation purposes). JARVIS training was performed with three different strategies: **a)** random cross-validation splits **b)** using a training set of ClinVar pathogenic and denovodb benign variants matched in terms of their distances to closest TSS and **c)** cross-validation splits stratified by chromosome (eigenPC did not have sufficient number of data points with a score for strategies *b* and *c* and thus is not shown in these plots). The red dashed horizontal line marks the p=0.05 significance threshold [$y = \mathrm{Phred}(0.05)$]. Barplots in light purple colour that exceed the significance threshold of Phred(0.05) support that JARVIS significantly outperforms the respective score (JARVIS > Query_score). On the other hand, barplots in orange colour that exceed the significance threshold of Phred(0.05) support that JARVIS significantly *underperforms* the respective score (JARVIS < Query_score). Raw p-values from each DeLong test are available at Supplementary Tables 4-6.

JARVIS: Cross-validation performance on four validation sets

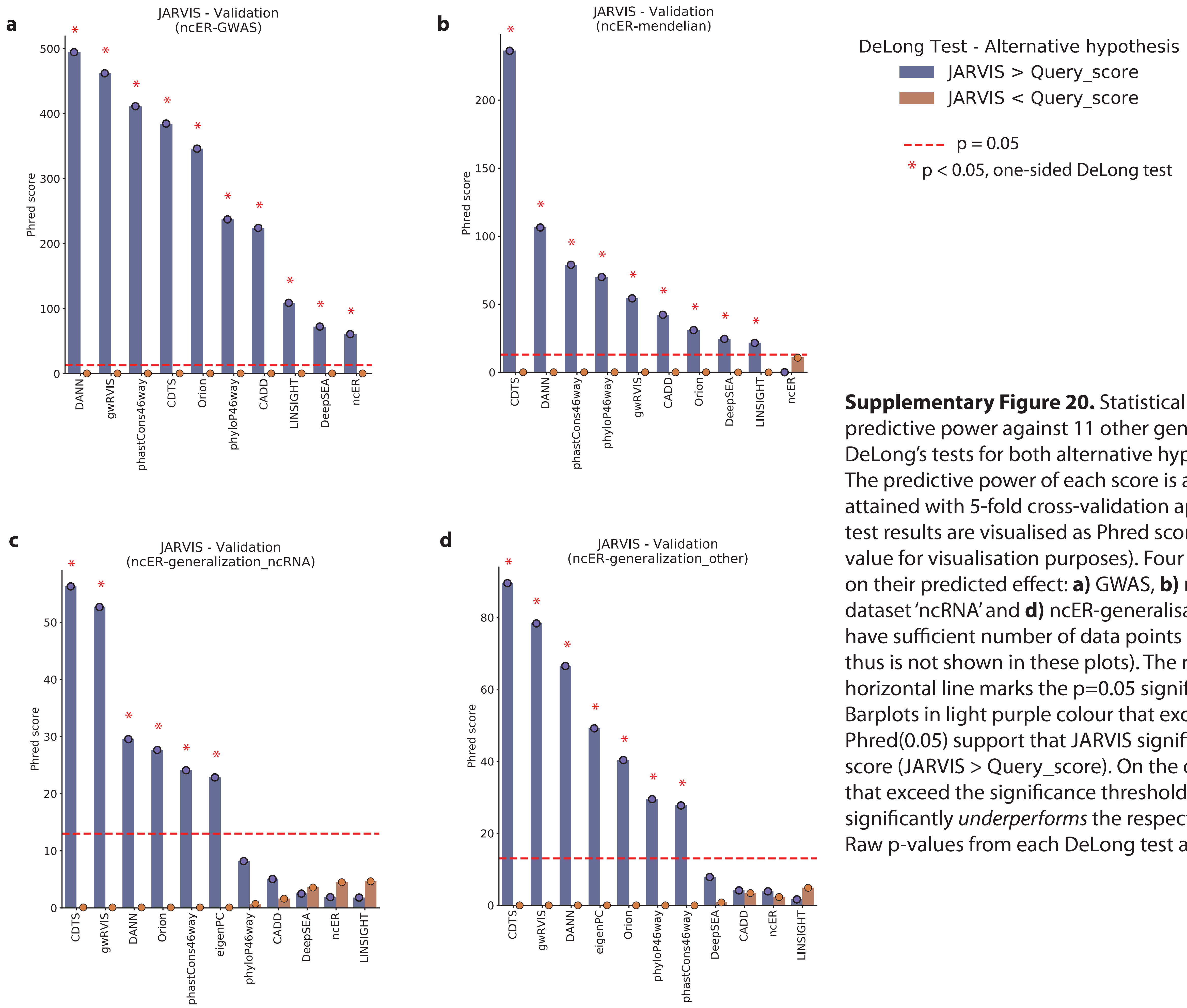

Supplementary Figure 20. Statistical significance of the differences in JARVIS predictive power against 11 other genome-wide scores using one-sided DeLong's tests for both alternative hypotheses (i.e. "less" or "greater"). The predictive power of each score is assessed via the average AUC score attained with 5-fold cross-validation applied on each validation set. DeLong's test results are visualised as Phred scores (scores > 500 are capped at this value for visualisation purposes). Four validation sets have been used based on their predicted effect: **a)** GWAS, **b)** mendelian, **c)** ncER-generalisation dataset 'ncRNA' and **d)** ncER-generalisation dataset 'other' (eigenPC did not have sufficient number of data points with a score for strategies *a* and *b* and thus is not shown in these plots). The red dashed horizontal line marks the p=0.05 significance threshold [y=Phred(0.05)]. Barplots in light purple colour that exceed the significance threshold of Phred(0.05) support that JARVIS significantly outperforms the respective score (JARVIS > Query_score). On the other hand, barplots in orange colour that exceed the significance threshold of Phred(0.05) support that JARVIS significantly *underperforms* the respective score (JARVIS < Query_score). Raw p-values from each DeLong test are available at Supplementary Tables 7-10.

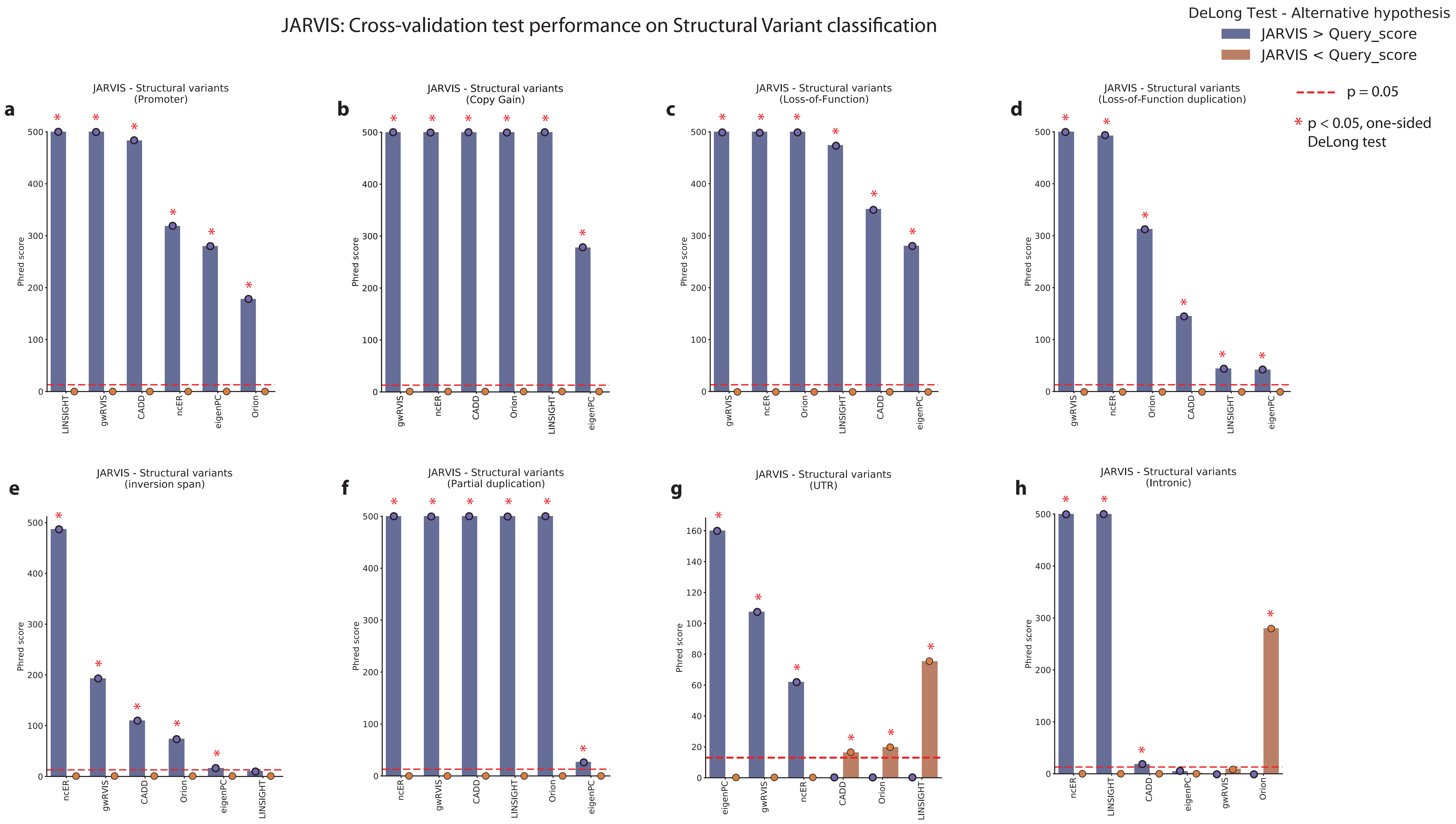

Supplementary Figure 21. Statistical significance of the differences in JARVIS predictive power against 6 other genome-wide scores for structrual variant classification using one-sided DeLong's tests for both alternative hypotheses (i.e. "less" or "greater"). The predictive power of each score is assessed via the average AUC score attained with 3-fold cross-validation when classifying each test set of structural variants against a control set of structural variants with intergenic effect. DeLong's test results are visualised as Phred scores (scores > 500 are capped at this value for visualisation purposes). 8 test sets of structrual variants have been used based on their predicted effect: **a)** Promoter, **b)** Copy Gain, **c)** Loss-of-Function, **d)** Loss-of-Function duplication, **e)** Inversion span, **f)** Partial duplication, **g)** UTR and **h)** Intronic. The red dashed horizontal line marks the p=0.05 significance threshold [$y=\mathrm{Phred}(0.05)$]. Barplots in light purple colour that exceed the significance threshold of Phred(0.05) support that JARVIS significantly outperforms the respective score (JARVIS > Query_score). On the other hand, barplots in orange colour that exceed the significance threshold of Phred(0.05) support that JARVIS significantly *underperforms* the respective score (JARVIS < Query_score). Raw p-values from each DeLong test are available at Supplementary Tables 11-18.

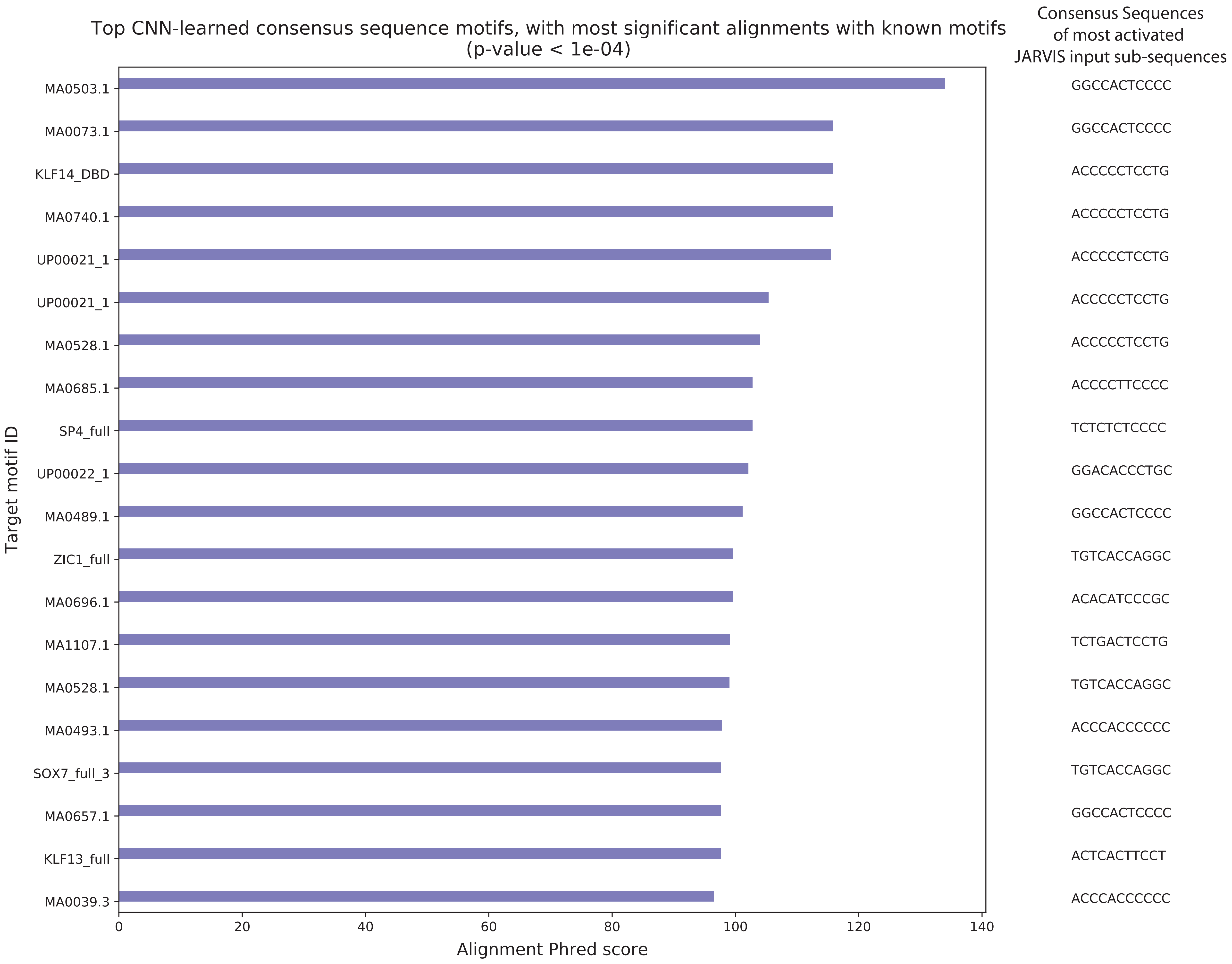

Supplementary Figure 22. Consensus sequences of most activated sequence clusters learnt by JARVIS-CNN module that most significantly align with known regulatory motifs.

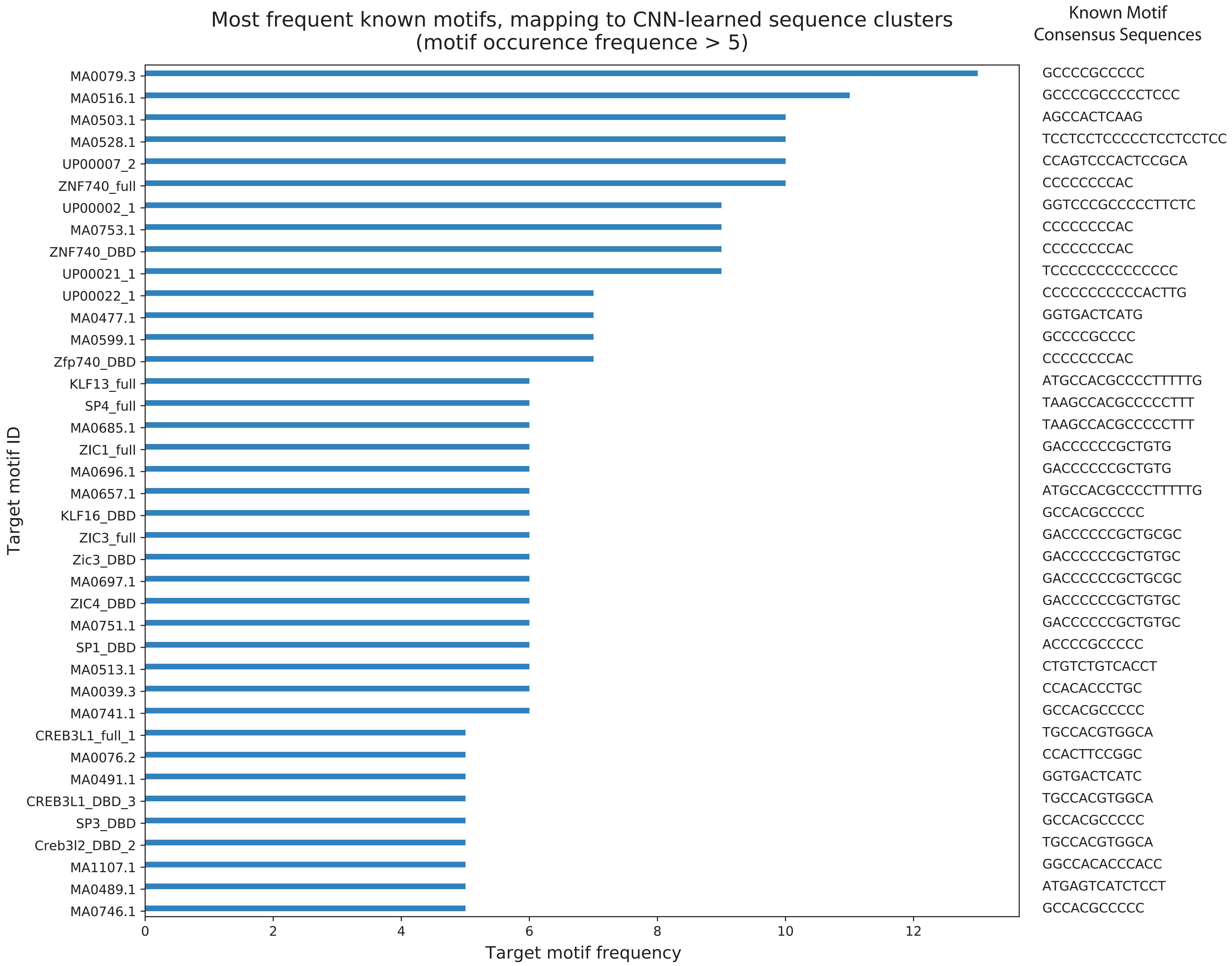

Supplementary Figure 23. Consensus sequences of most frequent known motifs extracted with TomTom (MEME Suite), mapping to the top sequence clusters learnt by JARVIS-CNN module.

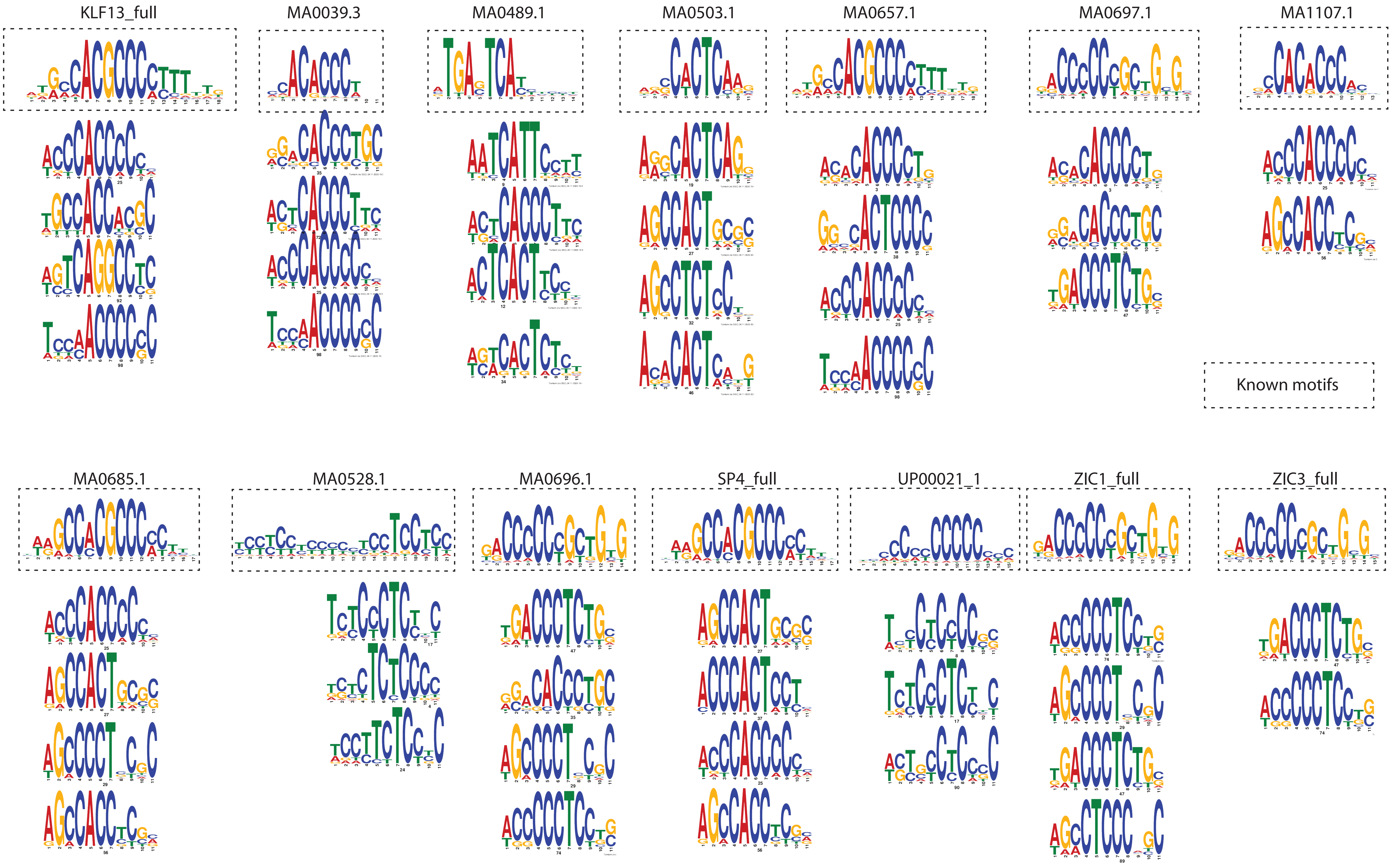

Supplementary Figure 24. Sequence logos of sequence clusters learnt by JARVIS-CNN module: intersection of known motifs that have most significantly aligned with JARVIS learnt sequence clusters having the highest activation sum. Known motifs are enclosed in rectangles with dashed lines, while motifs learnt by JARVIS are stacked below the respective known motif achieving the highest degree of alignment.

Supplementary Table 1. DeLong test results - gwRVIS cross-validation (lincRNAs). The p-value of the most significant one-sided DeLong test for each pairwise score comparison is shown in bold, as long as p < 0.05.

DeLong Test - Alternative hypothesis	Query_score	pval	Phred score
gwRVIS > Query_score	CADD	0.389058791	4.099847676
gwRVIS < Query_score	CADD	0.610941209	2.140005797
gwRVIS > Query_score	phyloP46way	**5.83E-03**	22.34364447
gwRVIS < Query_score	phyloP46way	0.994170443	2.54E-02
gwRVIS > Query_score	phastCons46way	**0.001749402**	2.76E+01
gwRVIS < Query_score	phastCons46way	9.98E-01	0.007604209
gwRVIS > Query_score	Orion	**1.20E-02**	19.20305451
gwRVIS < Query_score	Orion	0.987985809	0.052492936
gwRVIS > Query_score	gwRVIS + CADD	0.645707235	1.899643472
gwRVIS < Query_score	gwRVIS + CADD	0.354292765	4.506377166

Supplementary Table 2. DeLong test results - gwRVIS cross-validation (intergenic regions). The p-value of the most significant one-sided DeLong test for each pairwise score comparison is shown in bold, as long as p < 0.05.

DeLong Test - Alternative hypothesis	Query_score	pval	Phred score
gwRVIS > Query_score	CADD	0.343998126	4.634439236
gwRVIS < Query_score	CADD	0.656001874	1.830949198
gwRVIS > Query_score	phyloP46way	**2.65E-06**	55.76556991
gwRVIS < Query_score	phyloP46way	0.999997349	1.15E-05
gwRVIS > Query_score	phastCons46way	**0.00619332**	2.21E+01
gwRVIS < Query_score	phastCons46way	9.94E-01	0.026980883
gwRVIS > Query_score	Orion	**2.00E-02**	17.00039088
gwRVIS < Query_score	Orion	0.980049173	0.087521337
gwRVIS > Query_score	gwRVIS + CADD	0.594911204	2.255478517
gwRVIS < Query_score	gwRVIS + CADD	0.405088796	3.924497687

Supplementary Table 3. DeLong test results - gwRVIS cross-validation (UTRs). The p-value of the most significant one-sided DeLong test for each pairwise score comparison is shown in bold, as long as p < 0.05.

DeLong Test - Alternative hypothesis	Query_score	pval	Phred score
gwRVIS > Query_score	CADD	0.997140221	0.012437655
gwRVIS < Query_score	CADD	**0.002859779**	25.43667536
gwRVIS > Query_score	phyloP46way	9.53E-01	0.211017908
gwRVIS < Query_score	phyloP46way	**0.047427128**	1.32E+01
gwRVIS > Query_score	phastCons46way	**0.004104436**	2.39E+01
gwRVIS < Query_score	phastCons46way	9.96E-01	0.017862023
gwRVIS > Query_score	Orion	**4.71E-04**	33.2713895

gwRVIS < Query_score	Orion	0.999529173	0.002045256
gwRVIS > Query_score	gwRVIS + CADD	1	1.15E-11
gwRVIS < Query_score	gwRVIS + CADD	**2.66E-12**	115.75733

Supplementary Table 4. DeLong test results - JARVIS training: randomised cross-validation fold splits. The p-value of the most significant one-sided DeLong test for each pairwise score comparison is shown in bold, as long as p < 0.05.

DeLong Test - Alternative hypothesis	Query_score	pval	Phred score
JARVIS > Query_score	DeepSEA	0.963057336	0.163478561
JARVIS < Query_score	DeepSEA	0.063691255	11.95920193
JARVIS > Query_score	eigenPC	**9.29E-16**	150.3206768
JARVIS < Query_score	eigenPC	1	4.34E-15
JARVIS > Query_score	ncER	0.999979922	8.72E-05
JARVIS < Query_score	ncER	**2.01E-05**	46.97273414
JARVIS > Query_score	CDTS	**4.18E-102**	1013.784896
JARVIS < Query_score	CDTS	1	0
JARVIS > Query_score	LINSIGHT	0.976308745	0.104128206
JARVIS < Query_score	LINSIGHT	**0.023691255**	16.25411931
JARVIS > Query_score	gwRVIS	**5.68E-19**	182.4575293
JARVIS < Query_score	gwRVIS	1	0
JARVIS > Query_score	CADD	**0.000270548**	35.67755292
JARVIS < Query_score	CADD	0.999729452	0.001175135
JARVIS > Query_score	DANN	**4.00E-29**	283.9767252
JARVIS < Query_score	DANN	1	0
JARVIS > Query_score	phyloP46way	**5.80E-18**	172.3653734
JARVIS < Query_score	phyloP46way	1	0
JARVIS > Query_score	phastCons46way	**5.29E-43**	422.7643728
JARVIS < Query_score	phastCons46way	1	0
JARVIS > Query_score	Orion	**3.32E-16**	154.7839623
JARVIS < Query_score	Orion	1	0

Supplementary Table 5. DeLong test results - JARVIS training: matched variant distances from closest TSS. The p-value of the most significant one-sided DeLong test for each pairwise score comparison is shown in bold, as long as p < 0.05.

DeLong Test - Alternative hypothesis	Query_score	pval	Phred score
JARVIS > Query_score	DeepSEA	**0.00281974**	25.49790913
JARVIS < Query_score	DeepSEA	0.99718026	0.012263274
JARVIS > Query_score	ncER	1.00E+00	0.000214146
JARVIS < Query_score	ncER	**4.93E-05**	4.31E+01
JARVIS > Query_score	CDTS	**2.25E-23**	2.26E+02
JARVIS < Query_score	CDTS	1.00E+00	0
JARVIS > Query_score	LINSIGHT	9.24E-01	0.344950539

JARVIS < Query_score	LINSIGHT	0.087763553	10.56685804
JARVIS > Query_score	gwRVIS	**0.007073928**	21.50339379
JARVIS < Query_score	gwRVIS	0.992926072	0.030830855
JARVIS > Query_score	CADD	**2.14E-02**	16.70139926
JARVIS < Query_score	CADD	0.978627266	0.093826882
JARVIS > Query_score	DANN	**9.35E-07**	60.28969621
JARVIS < Query_score	DANN	0.999999065	4.06E-06
JARVIS > Query_score	phyloP46way	**3.71E-02**	14.30338096
JARVIS < Query_score	phyloP46way	0.96287539	0.164299134
JARVIS > Query_score	phastCons46way	**3.37E-12**	114.7259156
JARVIS < Query_score	phastCons46way	1	1.46E-11
JARVIS > Query_score	Orion	**3.03E-23**	225.188548
JARVIS < Query_score	Orion	1	0

Supplementary Table 6. DeLong test results - JARVIS training: cross-validation fold splits stratified by chromosome. The p-value of the most significant one-sided DeLong test for each pairwise score comparison is shown in bold, as long as p < 0.05.

**DeLong Test - Alternative hypothesis**	**Query_score**	**pval**	**Phred score**
JARVIS > Query_score	DeepSEA	0.06487824	11.87900938
JARVIS < Query_score	DeepSEA	0.98512176	0.06510088
JARVIS > Query_score	eigenPC	**4.04E-15**	143.9341466
JARVIS < Query_score	eigenPC	1.00E+00	1.74E-14
JARVIS > Query_score	ncER	1.00E+00	5.05E-08
JARVIS < Query_score	ncER	**1.16E-08**	79.34167227
JARVIS > Query_score	CDTS	**4.08E-91**	903.8926048
JARVIS < Query_score	CDTS	1	0
JARVIS > Query_score	LINSIGHT	0.999798139	0.000876759
JARVIS < Query_score	LINSIGHT	**0.000201861**	36.94947894
JARVIS > Query_score	gwRVIS	**3.64E-23**	224.3839185
JARVIS < Query_score	gwRVIS	1	0
JARVIS > Query_score	CADD	1.58E-01	8.015685548
JARVIS < Query_score	CADD	0.842082069	7.46E-01
JARVIS > Query_score	DANN	**3.20E-16**	154.9528648
JARVIS < Query_score	DANN	1	0
JARVIS > Query_score	phyloP46way	**9.05E-13**	120.4344869
JARVIS < Query_score	phyloP46way	1	3.93E-12
JARVIS > Query_score	phastCons46way	**6.77E-33**	321.6942123
JARVIS < Query_score	phastCons46way	1	0
JARVIS > Query_score	Orion	**5.50E-11**	102.5935685
JARVIS < Query_score	Orion	1	2.39E-10

Supplementary Table 7. DeLong test results - JARVIS Testing: ncER GWAS dataset (Wells et

al.). The p-value of the most significant one-sided DeLong test for each pairwise score comparison is shown in bold, as long as $p < 0.05$.

DeLong Test - Alternative hypothesis	Query_score	pval	Phred score
JARVIS > Query_score	DeepSEA	**5.56E-08**	72.55206268
JARVIS < Query_score	DeepSEA	0.999999944	2.41E-07
JARVIS > Query_score	ncER	**7.65E-07**	61.16227987
JARVIS < Query_score	ncER	0.999999235	3.32E-06
JARVIS > Query_score	CDTS	**3.39E-39**	3.85E+02
JARVIS < Query_score	CDTS	1.00E+00	0
JARVIS > Query_score	LINSIGHT	**1.25E-11**	109.0397495
JARVIS < Query_score	LINSIGHT	1	5.42E-11
JARVIS > Query_score	gwRVIS	**6.21E-47**	462.0659146
JARVIS < Query_score	gwRVIS	1	0
JARVIS > Query_score	CADD	**3.67E-23**	224.353134
JARVIS < Query_score	CADD	1	0
JARVIS > Query_score	DANN	**3.49E-50**	494.5659796
JARVIS < Query_score	DANN	1	0
JARVIS > Query_score	phyloP46way	**1.83E-24**	237.3664035
JARVIS < Query_score	phyloP46way	1	0
JARVIS > Query_score	phastCons46way	**7.43E-42**	411.2928909
JARVIS < Query_score	phastCons46way	1	0
JARVIS > Query_score	Orion	**2.44E-35**	346.1177985
JARVIS < Query_score	Orion	1	0

Supplementary Table 8. DeLong test results - JARVIS Testing: ncER Mendelian dataset (Wells et al.). The p-value of the most significant one-sided DeLong test for each pairwise score comparison is shown in bold, as long as $p < 0.05$.

DeLong Test - Alternative hypothesis	Query_score	pval	Phred score
JARVIS > Query_score	DeepSEA	**3.48E-03**	24.58799486
JARVIS < Query_score	DeepSEA	0.996523033	1.51E-02
JARVIS > Query_score	ncER	9.19E-01	0.367785998
JARVIS < Query_score	ncER	0.081199125	1.09E+01
JARVIS > Query_score	CDTS	**2.36E-24**	2.36E+02
JARVIS < Query_score	CDTS	1.00E+00	0
JARVIS > Query_score	LINSIGHT	**6.70E-03**	21.7378574
JARVIS < Query_score	LINSIGHT	0.993297848	2.92E-02
JARVIS > Query_score	gwRVIS	**3.62E-06**	54.41595365
JARVIS < Query_score	gwRVIS	0.999996383	1.57E-05
JARVIS > Query_score	CADD	**5.92E-05**	42.2796431
JARVIS < Query_score	CADD	0.999940839	0.000256941
JARVIS > Query_score	DANN	**2.30E-11**	106.3796977
JARVIS < Query_score	DANN	1	1.00E-10
JARVIS > Query_score	phyloP46way	**9.72E-08**	70.12433983

JARVIS < Query_score	phyloP46way	0.999999903	4.22E-07
JARVIS > Query_score	phastCons46way	**1.26E-08**	78.98242191
JARVIS < Query_score	phastCons46way	0.999999987	5.49E-08
JARVIS > Query_score	Orion	**8.07E-04**	30.9302414
JARVIS < Query_score	Orion	0.99919281	0.003506998

Supplementary Table 9. DeLong test results - JARVIS Testing: ncER generalisation _ncRNA_ dataset (Wells et al.). The p-value of the most significant one-sided DeLong test for each pairwise score comparison is shown in bold, as long as p < 0.05.

DeLong Test - Alternative hypothesis	Query_score	pval	Phred score
JARVIS > Query_score	DeepSEA	5.61E-01	2.509373556
JARVIS < Query_score	DeepSEA	0.43887109	3.58E+00
JARVIS > Query_score	eigenPC	**5.18E-03**	22.85628246
JARVIS < Query_score	eigenPC	0.994819499	2.26E-02
JARVIS > Query_score	ncER	6.45E-01	1.90E+00
JARVIS < Query_score	ncER	3.55E-01	4.500603917
JARVIS > Query_score	CDTS	**2.35E-06**	56.28791839
JARVIS < Query_score	CDTS	0.999997649	1.02E-05
JARVIS > Query_score	LINSIGHT	6.59E-01	1.813676509
JARVIS < Query_score	LINSIGHT	0.341383891	4.667569764
JARVIS > Query_score	gwRVIS	**5.39E-06**	52.68414642
JARVIS < Query_score	gwRVIS	0.99999461	2.34E-05
JARVIS > Query_score	CADD	3.12E-01	5.061042101
JARVIS < Query_score	CADD	0.688185871	1.622942479
JARVIS > Query_score	DANN	**1.11E-03**	29.53987756
JARVIS < Query_score	DANN	0.998888237	0.004831012
JARVIS > Query_score	phyloP46way	1.51E-01	8.202390762
JARVIS < Query_score	phyloP46way	0.848727173	0.712318933
JARVIS > Query_score	phastCons46way	**3.88E-03**	24.11516033
JARVIS < Query_score	phastCons46way	0.996123106	0.016869861

Supplementary Table 10. DeLong test results - JARVIS Testing: ncER generalisation _other_ dataset (Wells et al.). The p-value of the most significant one-sided DeLong test for each pairwise score comparison is shown in bold, as long as p < 0.05.

DeLong Test - Alternative hypothesis	Query_score	pval	Phred score
JARVIS > Query_score	DeepSEA	1.64E-01	7.847464935
JARVIS < Query_score	DeepSEA	0.83584523	7.79E-01
JARVIS > Query_score	eigenPC	**1.22E-05**	49.15063831
JARVIS < Query_score	eigenPC	0.99998784	5.28E-05
JARVIS > Query_score	ncER	4.11E-01	3.86E+00
JARVIS < Query_score	ncER	5.89E-01	2.302121763
JARVIS > Query_score	CDTS	**1.12E-09**	89.51273422

JARVIS < Query_score	CDTS	0.999999999	4.86E-09
JARVIS > Query_score	LINSIGHT	6.77E-01	1.693903499
JARVIS < Query_score	LINSIGHT	0.322967292	4.908414574
JARVIS > Query_score	gwRVIS	**1.45E-08**	78.38702157
JARVIS < Query_score	gwRVIS	0.999999986	6.30E-08
JARVIS > Query_score	CADD	3.82E-01	4.17892218
JARVIS < Query_score	CADD	0.447960928	3.487598648
JARVIS > Query_score	DANN	**2.21E-07**	66.55106582
JARVIS < Query_score	DANN	0.999999779	9.61E-07
JARVIS > Query_score	phyloP46way	**1.10E-03**	29.57657096
JARVIS < Query_score	phyloP46way	0.998897591	0.004790344
JARVIS > Query_score	phastCons46way	**1.66E-03**	27.78669444
JARVIS < Query_score	phastCons46way	0.998335321	0.007235634

Supplementary Table 11. DeLong test results - JARVIS Testing on Structural Variants (Promoter). The p-value of the most significant one-sided DeLong test for each pairwise score comparison is shown in bold, as long as $p < 0.05$.

DeLong Test - Alternative hypothesis	Query_score	pval	Phred score
JARVIS > Query_score	ncER	**1.36E-32**	318.6787464
JARVIS < Query_score	ncER	1	0
JARVIS > Query_score	LINSIGHT	**1.22E-104**	1039.135936
JARVIS < Query_score	LINSIGHT	1	0.00E+00
JARVIS > Query_score	gwRVIS	**6.59E-64**	6.32E+02
JARVIS < Query_score	gwRVIS	1.00E+00	0
JARVIS > Query_score	CADD	**4.76E-49**	483.2272732
JARVIS < Query_score	CADD	1	0
JARVIS > Query_score	eigen	**9.61E-29**	280.170654
JARVIS < Query_score	eigen	1	0
JARVIS > Query_score	Orion	**1.50E-18**	178.225197
JARVIS < Query_score	Orion	1	0

Supplementary Table 12. DeLong test results - JARVIS Testing on Structural Variants (Copy Gain). The p-value of the most significant one-sided DeLong test for each pairwise score comparison is shown in bold, as long as $p < 0.05$.

DeLong Test - Alternative hypothesis	Query_score	pval	Phred score
JARVIS > Query_score	ncER	**3.39E-192**	1914.696973
JARVIS < Query_score	ncER	1	0
JARVIS > Query_score	LINSIGHT	**8.26E-73**	720.8277866
JARVIS < Query_score	LINSIGHT	1	0.00E+00
JARVIS > Query_score	gwRVIS	**< 1e-308**	3.08E+03
JARVIS < Query_score	gwRVIS	1.00E+00	0

JARVIS > Query_score	CADD	**8.19E-152**	1510.865332
JARVIS < Query_score	CADD	1	0
JARVIS > Query_score	eigen	**1.59E-28**	277.9789233
JARVIS < Query_score	eigen	1	0
JARVIS > Query_score	Orion	**1.09E-134**	1339.620113
JARVIS < Query_score	Orion	1	0

Supplementary Table 13. DeLong test results - JARVIS Testing on Structural Variants (Loss-of-Function). The p-value of the most significant one-sided DeLong test for each pairwise score comparison is shown in bold, as long as $p < 0.05$.

DeLong Test - Alternative hypothesis	Query_score	pval	Phred score
JARVIS > Query_score	ncER	**3.04E-192**	1915.164141
JARVIS < Query_score	ncER	1	0
JARVIS > Query_score	LINSIGHT	**3.73E-48**	474.286569
JARVIS < Query_score	LINSIGHT	1	0.00E+00
JARVIS > Query_score	gwRVIS	**< 1e-308**	3.08E+03
JARVIS < Query_score	gwRVIS	1.00E+00	0
JARVIS > Query_score	CADD	**7.00E-36**	351.5508806
JARVIS < Query_score	CADD	1	0
JARVIS > Query_score	eigen	**9.24E-29**	280.3432965
JARVIS < Query_score	eigen	1	0
JARVIS > Query_score	Orion	**1.73E-137**	1367.61491
JARVIS < Query_score	Orion	1	0

Supplementary Table 14. DeLong test results - JARVIS Testing on Structural Variants (Loss-of-Function Duplication). The p-value of the most significant one-sided DeLong test for each pairwise score comparison is shown in bold, as long as $p < 0.05$.

DeLong Test - Alternative hypothesis	Query_score	pval	Phred score
JARVIS > Query_score	ncER	**5.23E-50**	492.8158561
JARVIS < Query_score	ncER	1	0
JARVIS > Query_score	LINSIGHT	**3.81E-05**	44.18767303
JARVIS < Query_score	LINSIGHT	0.999961873	1.66E-04
JARVIS > Query_score	gwRVIS	**1.43E-55**	5.48E+02
JARVIS < Query_score	gwRVIS	1.00E+00	0
JARVIS > Query_score	CADD	**2.91E-15**	145.3602313
JARVIS < Query_score	CADD	1	1.30E-14
JARVIS > Query_score	eigen	**6.14E-05**	42.12100307
JARVIS < Query_score	eigen	0.999938638	0.0002665
JARVIS > Query_score	Orion	**4.99E-32**	313.0178188
JARVIS < Query_score	Orion	1	0

Supplementary Table 15. DeLong test results - JARVIS Testing on Structural Variants (Inversion span). The p-value of the most significant one-sided DeLong test for each pairwise score comparison is shown in bold, as long as p < 0.05.

DeLong Test - Alternative hypothesis	Query_score	pval	Phred score
JARVIS > Query_score	ncER	**1.91E-49**	487.1830924
JARVIS < Query_score	ncER	1	0
JARVIS > Query_score	LINSIGHT	**8.72E-02**	10.59317933
JARVIS < Query_score	LINSIGHT	0.912766747	3.96E-01
JARVIS > Query_score	gwRVIS	**4.90E-20**	1.93E+02
JARVIS < Query_score	gwRVIS	1.00E+00	0
JARVIS > Query_score	CADD	**9.18E-12**	110.3722289
JARVIS < Query_score	CADD	1	3.99E-11
JARVIS > Query_score	eigen	**2.19E-02**	16.60316126
JARVIS < Query_score	eigen	0.978138303	0.095997343
JARVIS > Query_score	Orion	**3.86E-08**	74.13388676
JARVIS < Query_score	Orion	0.999999961	1.68E-07

Supplementary Table 16. DeLong test results - JARVIS Testing on Structural Variants (Partial Duplication). The p-value of the most significant one-sided DeLong test for each pairwise score comparison is shown in bold, as long as p < 0.05.

DeLong Test - Alternative hypothesis	Query_score	pval	Phred score
JARVIS > Query_score	ncER	**3.04E-296**	2955.171889
JARVIS < Query_score	ncER	1	0
JARVIS > Query_score	LINSIGHT	**4.38E-155**	1543.583129
JARVIS < Query_score	LINSIGHT	1	0.00E+00
JARVIS > Query_score	gwRVIS	**2.44E-269**	2.69E+03
JARVIS < Query_score	gwRVIS	1.00E+00	0
JARVIS > Query_score	CADD	**3.62E-159**	1584.414764
JARVIS < Query_score	CADD	1	0
JARVIS > Query_score	eigen	**2.05E-03**	26.8875657
JARVIS < Query_score	eigen	0.997952408	0.008901696
JARVIS > Query_score	Orion	**6.42E-108**	1071.921546
JARVIS < Query_score	Orion	1	0

Supplementary Table 17. DeLong test results - JARVIS Testing on Structural Variants (UTR). The p-value of the most significant one-sided DeLong test for each pairwise score comparison is shown in bold, as long as p < 0.05.

DeLong Test - Alternative hypothesis	Query_score	pval	Phred score
JARVIS > Query_score	ncER	**6.12E-07**	62.13408916
JARVIS < Query_score	ncER	0.999999388	2.66E-06

JARVIS > Query_score	LINSIGHT	1.00E+00	1.21E-07
JARVIS < Query_score	LINSIGHT	**2.80E-08**	7.55E+01
JARVIS > Query_score	gwRVIS	**1.78E-11**	1.08E+02
JARVIS < Query_score	gwRVIS	1.00E+00	7.71E-11
JARVIS > Query_score	CADD	9.78E-01	0.097790789
JARVIS < Query_score	CADD	**0.022265542**	16.52366726
JARVIS > Query_score	eigen	**9.55E-17**	160.200891
JARVIS < Query_score	eigen	1	0
JARVIS > Query_score	Orion	0.989780274	0.04461206
JARVIS < Query_score	Orion	**1.02E-02**	19.90560727

Supplementary Table 18. DeLong test results - JARVIS Testing on Structural Variants (Intronic). The p-value of the most significant one-sided DeLong test for each pairwise score comparison is shown in bold, as long as p < 0.05.

DeLong Test - Alternative hypothesis	Query_score	pval	Phred score
JARVIS > Query_score	ncER	**9.81E-217**	2160.082503
JARVIS < Query_score	ncER	1	0
JARVIS > Query_score	LINSIGHT	**1.70E-94**	937.6896328
JARVIS < Query_score	LINSIGHT	1	0.00E+00
JARVIS > Query_score	gwRVIS	8.90E-01	5.05E-01
JARVIS < Query_score	gwRVIS	1.10E-01	9.59386659
JARVIS > Query_score	CADD	**1.44E-02**	18.42102034
JARVIS < Query_score	CADD	0.985615394	0.06292522
JARVIS > Query_score	eigen	3.04E-01	5.177135734
JARVIS < Query_score	eigen	0.696410724	1.571345499
JARVIS > Query_score	Orion	1.00E+00	0
JARVIS < Query_score	Orion	**9.86E-29**	280.0598626
