# Supplementary material for: Prioritizing non-coding regions based on human genomic constraint and sequence context with deep learning
Source: Nat Commun. 2021 Mar 8;12:1504. doi: 10.1038/s41467-021-21790-4 (PMC7940646; doi:10.1038/s41467-021-21790-4)
Supplement: Supplementary file 3 — Description of Additional Supplementary Files [file 41467_2021_21790_MOESM3_ESM.pdf]

Description of Additional Supplementary Files

File name: Supplementary Data 1.

Description: Top JARVIS-learnt sequence clusters aligning with known motifs derived with the MEME Suite of tools.
